# Distinct Thalamo‐Subcortical Circuits Underlie Painful Behavior and Depression‐Like Behavior Following Nerve Injury

**DOI:** 10.1002/advs.202401855

**Published:** 2024-07-07

**Authors:** Jie Deng, Li Chen, Cui‐Cui Liu, Meng Liu, Guo‐Qing Guo, Jia‐You Wei, Jian‐Bo Zhang, Hai‐Ting Fan, Zi‐Kun Zheng, Pu Yan, Xiang‐Zhong Zhang, Feng Zhou, Sui‐Xiang Huang, Ji‐Feng Zhang, Ting Xu, Jing‐Dun Xie, Wen‐Jun Xin

**Affiliations:** ^1^ Department of Physiology and Pain Research Center Neuroscience Program Zhongshan School of Medicine The Fifth Affiliated Hospital Guangdong Province Key Laboratory of Brain Function and Disease Sun Yat‐Sen University Guangzhou 510080 China; ^2^ Guangdong Provincial Key Laboratory of Malignant Tumor Epigenetics and Gene Regulation Department of Rehabilitation Medicine Sun Yat‐Sen Memorial Hospital Sun Yat‐Sen University Guangzhou 510120 China; ^3^ Department of Anesthesia and Pain Medicine Guangzhou First People's Hospital Guangzhou 510000 China; ^4^ Neuroscience Laboratory for Cognitive and Developmental Disorders Department of Anatomy Medical College of Jinan University Guangzhou 510630 China; ^5^ Department of Pain Medicine The State Key Clinical Specialty in Pain Medicine The Second Affiliated Hospital Guangzhou Medical University Guangzhou 510630 China; ^6^ Department of Anesthesiology The First Affiliated Hospital Sun Yat‐sen University Guangzhou 510080 China; ^7^ Department of Electronic Engineering Shantou University Shantou 515063 China; ^8^ Department of Hematology The Third Affiliated Hospital of Sun Yat‐sen University Guangzhou 510630 China; ^9^ Department of Neurology First people's hospital of Foshan Foshan Guangdong 510168 China; ^10^ Department of Pain Medicine Guangzhou Red Cross Hospital Affiliated to Jinan University Guangzhou 510630 China; ^11^ State Key Laboratory of Oncology in Southern China Collaborative Innovation for Cancer Medicine Sun Yat‐sen University Cancer Center Guangzhou 510060 China

**Keywords:** chronic pain, comorbidity, depression, nucleus accumbens, paraventricular thalamus anterior, paraventricular thalamus posterior, ventrolateral periaqueductal gray

## Abstract

Clinically, chronic pain and depression often coexist in multiple diseases and reciprocally reinforce each other, which greatly escalates the difficulty of treatment. The neural circuit mechanism underlying the chronic pain/depression comorbidity remains unclear. The present study reports that two distinct subregions in the paraventricular thalamus (PVT) play different roles in this pathological process. In the first subregion PVT posterior (PVP), glutamatergic neurons (PVP^Glu^) send signals to GABAergic neurons (VLPAG^GABA^) in the ventrolateral periaqueductal gray (VLPAG), which mediates painful behavior in comorbidity. Meanwhile, in another subregion PVT anterior (PVA), glutamatergic neurons (PVA^Glu^) send signals to the nucleus accumbens D1‐positive neurons and D2‐positive neurons (NAc^D1→D2^), which is involved in depression‐like behavior in comorbidity. This study demonstrates that the distinct thalamo‐subcortical circuits PVP^Glu^→VLPAG^GABA^ and PVA^Glu^→NAc^D1→D2^ mediated painful behavior and depression‐like behavior following spared nerve injury (SNI), respectively, which provides the circuit‐based potential targets for preventing and treating comorbidity.

## Graphical abstract

The data suggests that two subregions of the paraventricular thalamus have different activities and distinct roles in the comorbidity of chronic pain and depression. Following nerve injury, enhanced excitatory of the posterior paraventricular thalamus‐ventrolateral periaqueductal gray circuit is involved in the painful behavior in comorbid. Decreased excitatory of the anterior paraventricular thalamus‐nucleus accumbens circuit contributed to the depression‐like behavior in comorbid.

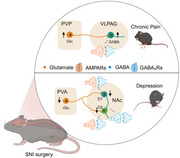

## 1 Introduction

Epidemiological studies show that up to 52% of patients with chronic pain exhibit depression, while up to 65% of depression patients are afflicted by pain symptoms.^[^ ^1^ ^]^ Chronic pain and depression interact with and reinforce each other, greatly exacerbating the difficulty of treatment in multiple pertinent diseases.^[^ ^2^ ^]^ Studies reveal that brain circuits are closely related to the development of chronic pain or depression.^[^ ^3^ ^]^ However, the brain circuits mediating the chronic pain/depression comorbidity remains unclear. Therefore, elucidating the brain circuit underlying the comorbidity of chronic pain/depression provides the potential effective therapeutic targets.

The thalamus is a key relaying hub for the transmission of nociceptive information.^[^ ^4^ ^]^ The paraventricular thalamus (PVT), as a part of the dorsal midline thalamus, is primarily composed of excitatory neurons.^[^ ^5^ ^]^ Accumulating evidence shows that PVT is involved in arousal, emotional valence, internal physiological state, and associative memory.^[^ ^5^, ^6^ ^]^ Recently, our and peer's study showed that nerve injury‐induced the chronic pain/depression comorbidity in rodents.^[^ ^7^ ^]^ However, it is currently not reported whether and how PVT participates in the comorbidity behavior.

Evidence from tracing and anatomy has shown that PVT receives input from a wide variety of cortical and subcortical areas involved in reward and stress‐related behavior. For example, prelimbic cortex glutamatergic projections to the PVT modulated the motivated behaviors through formation and maintenance of the association between cues and aversive or appetitive stimuli.^[^ ^8^ ^]^ In addition, the PVT also sends glutamatergic efferent to many brain regions including bed nucleus of the stria terminalis (BNST), hypothalamus, nucleus accumbens (NAc), periaqueductal gray (PAG), and amygdala, and is associated with arousal, stress, anxiety, and fear.^[^ ^9^ ^]^ Structurally, PVT is often divided into the anterior (PVA) and posterior (PVP) subregions based on anatomical boundaries that correspond with observational differences in cellular organization and function.^[^ ^10^ ^]^ Functionally, studies show that PVA may participate in the expression of approach behaviors. For example, inhibition of PVA played an important role in seeking behavior for sucrose or cocaine.^[^ ^11^ ^]^ Meanwhile, the PVP is important for avoidance behavior responsive to chronic stress.^[^ ^12^ ^]^ Recent research suggested that the different neural circuits involving PVT subregions (PVA and PVP) may mediate distinct biological functions. For example, the circuit of PVA→SCN (the suprachiasmatic nucleus) was related to circadian rhythm, while the projection from PVP to the amygdala was involved in the fear.^[^ ^9^, ^13^ ^]^ Currently, it is unclear regarding the role of PVT‐related neural circuits in the chronic pain/depression comorbidity setting.

In the present study, we used in vivo calcium photometry recording and in vitro electrophysiological recordings to present the adaption and function of the PVP and PVA neurons in spared nerve injury (SNI)‐induced comorbidity. Selectively manipulating the activity of glutamatergic neurons in PVP or PVA regulated the chronic pain and depression‐like behavior, respectively, in the mice following SNI. Furthermore, we dissected the fine circuit of PVP**→**VLPAG and PVA→NAc and found that differential activity of PVP^Glu^→VLPAG^GABA^ and PVA^Glu^→NAc^D1→D2^ mediated the painful behavior and depression‐like behavior in comorbidity mice, respectively.

## 2 Results

### 2.1 Different Activity of the PVA and the PVP in Comorbidity Mice Following SNI

**Figure 1:**
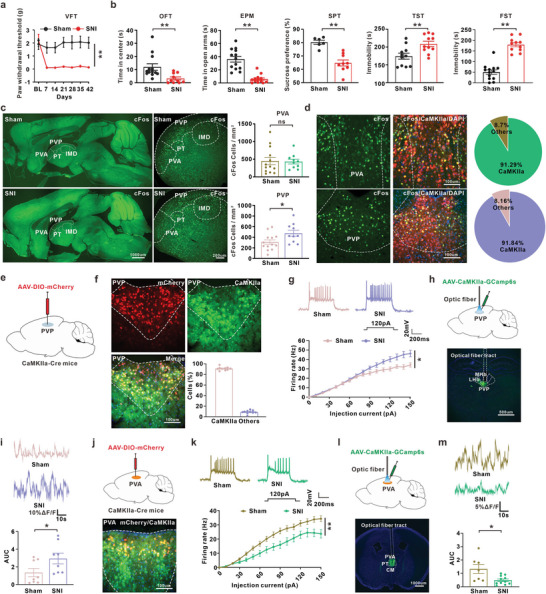
The hyper‐excitability of PVPGlu and hypo‐excitability of PVAGlu were observed in the SNI‐induced comorbidity mice. a) SNI induced a persistent mechanical allodynia (Sham: n = 6 mice; SNI: n = 6 mice; F(1,10) = 44.68, p < 0.0001). b) Depression‐like behaviors including OFT, EPM, SPT, TST and FST were performed in week 6 in mice (OFT: Sham, n = 13 mice; SNI, n = 10 mice; U = 18, p = 0.0025; EPM: Sham, n = 13 mice; SNI, n = 13 mice; U = 3.5, p < 0.0001; SPT: Sham, n = 6 mice; SNI, n = 9 mice; t(13) = 5.092, p = 0.0002; TST: Sham, n = 11 mice; SNI, n = 11 mice; t(20) = 3.061, p = 0.0062; FST: Sham, n = 13 mice; SNI, n = 11 mice; t(22) = 8.871, p < 0.0001). c) Left: from sagittal slice, cFos expression was increased in PVP, but not PVA, in comorbidity mice. Scale bar, 1000 um. Right: the magnified images. Scale bar, 200 um (PVA: Sham, n = 13 mice; SNI, n = 10 mice; U = 55, p = 0.5629; PVP: Sham, n = 13 mice; SNI, n = 10 mice; t(21) = 2.381, p = 0.0268). d) Left: the immunostaining results for the coronal section showed that the expression of cFos in PVA and PVP was colocalized with CaMKIIa‐positive neurons. Scale bar, 100 um. Right: quantitative analysis of the colocalization (n = 12 images from 3 mice). e) Schema of PVP with AAV‐DIO‐mCherry in CaMKIIa‐Cre mice. f) Typical images and quantitative analysis of immunofluorescence showed the colocalization of mCherry‐positive cells with CaMKIIa‐positive neurons following intra‐PVP injection of AAV‐DIO‐mCherry in CaMKIIa‐Cre mice. Scale bar, 100 um (n = 10 images from 3 mice). g) Representative trace (upper) and statistical data (lower) for the action potential firing recorded from PVPGlu neurons in week 6 following SNI or sham (Sham, n = 25 cells from 5 mice; SNI, n = 20 cells from 4 mice; F(1, 43) = 4.968, p = 0.0311). h) Scheme for the location of AAV‐CaMKIIa‐Gcamp6s injection (upper), typical image of viral expression and optical fiber in PVP of mice (lower). Scale bar, 500 um. i) Example of fiber photometry traces (upper) and quantitative analysis for histogram of area under the curve (AUC) of fiber photometry traces (lower) from PVPGlu neurons in mice treated with sham or SNI (Sham, n = 8 mice; SNI, n = 8 mice; U = 12, p = 0.0379). j) Schema of PVA injection with AAV‐DIO‐mCherry in CaMKIIa‐Cre mice (upper). Typical immunofluorescence images of the injection site and viral expression was co‐located with CaMKIIa‐positive neurons (lower). Scale bar, 100 um. k) Representative traces (upper) and statistical data (lower) for the action potential firing recorded from PVAGlu neurons on week 6 in mice treated with sham or SNI (Sham, n = 30 cells from 5 mice; SNI, n = 24 cells from 4 mice; F(1, 52) = 7.361, p = 0.009). l) Schema of the location of AAV‐CaMKIIa‐GCamp6s injection (upper), typical image of viral expression and optical fiber in PVA of mice (lower). Scale bar, 1000 um. m) Example (upper) and histogram of area under the curve (AUC) (lower) of fiber photometry traces from PVAGlu neurons in mice treated with sham or SNI (Sham, n = 7 mice; SNI, n = 11 mice; t(16) = 2.833, p = 0.012). All data were presented as the mean ± s.e.m. *P < 0.05, **P < 0.01. For detailed statistical information, see Supporting Table.

Consistent with our previous study,^[^ ^7a^ ^]^ a comorbidity model of chronic pain/depression‐like behavior was established in mice following spared nerve injury (SNI) (Figure S1a, Supporting Information). The Von Frey test (VFT) and depression‐like behavior testing, including open field test (OFT), elevated plus maze test (EPM), sucrose preference test (SPT), tail suspension test (TST), and forced swimming test (FST), were performed according to experimental schedule to confirm the phenotype of chronic pain and depression (Figure S1b, Supporting Information). The results revealed that SNI induced significant mechanical allodynia in week 1, which persisted until the end of the experiment (**Figure** **1**a). In addition, SNI‐treated mice displayed multiple depression‐like behaviors, including the decreased time in center in OFT and in open arms in EPM, the reduced sucrose preference in SPT, and the increased immobility time in TST and FST in week 6 (Figure 1b).

Evidence showed that the paraventricular thalamus (PVT) was composed of glutamatergic neurons (CaMKIIa‐positive cells), so we first investigated the neuronal activity properties in PVT in comorbidity mice. Immunofluorescence staining of sagittal planes showed an obvious increase of cFos expression in the posterior of paraventricular thalamus (PVP), rather than the anterior of paraventricular thalamus (PVA), on week 6 following SNI (Figure 1c), and cFos signals of coronal planes in PVP and PVA were co‐localized with CaMKIIa‐positive neurons (Figure 1d), indicating an enhanced neuronal activity in PVP glutamatergic neurons (PVP^Glu^) in comorbidity mice. To further confirm the hyperexcitability of PVP^Glu^ in a comorbidity setting, we injected AAV‐DIO‐mCherry into the PVP of CaMKIIa‐Cre mice (Figure 1e). 91.04% mCherry+CaMKIIa‐positive cells were colocalized with mCherry‐positive cells, indicating the specificity and efficiency of AAV transfection (Figure 1f). Further whole‐cell recordings showed that the number of action potentials evoked by depolarizing currents was obviously increased in the labeled PVP^Glu^ neurons (mCherry‐positive cells) in SNI‐induced comorbid mice relative to the corresponding control group (Figure 1g). Furthermore, in the comorbidity mice receiving a PVP infusion of AAV expressing CaMKIIa and fluorescent Ca^2+^ signals indicator GCaMP6s (AAV‐CaMKIIa‐GCaMP6s), a robust calcium signal in PVP^Glu^ was recorded by using optical fiber photometer recording compared with the corresponding control group (Figure 1h,i). In addition, mechanical stimuli (2g‐von Frey filament) rapidly increased Ca^2+^ signal in PVP^Glu^ of naive mice and comorbid mice, and the increase in comorbid mice had greater content than that in naïve mice (Figure S1c–e, Supporting Information), but aversive stimuli including FST and TST did not change the Ca^2+^ signal in PVP^Glu^ of both naïve and comorbid mice (Figure S1f–i, Supporting Information). These results suggested that the enhanced excitability of PVP^Glu^ may be involved in the painful behavior in the SNI‐induced comorbidity mice.

We then measured the activity of glutamatergic neurons of PVA (PVA^Glu^) with the same viral injection strategy (Figure 1j). Whole‐cell patch recordings showed that the number of action potential evoked by depolarizing currents in PVA^Glu^ significantly decreased in comorbidity mice relative to the corresponding control group (Figure 1k). Following injection of AAV‐CaMKIIa‐GCaMP6s (Figure 1l), the calcium signal of PVA^Glu^ neurons was significantly decreased in comorbidity mice relative to the corresponding control group (Figure 1l,m). Furthermore, the stimulation of FST or TST significantly reduced the Ca^2+^ signal in PVA^Glu^ of naive mice and comorbid mice, and the decrease in comorbid mice was more significant than that in naïve mice, but mechanical stimuli did not change the Ca^2+^ signal in PVA^Glu^ in both naïve and comorbid mice (Supplementary Figure 1j–m). These results suggested that the decreased PVA^Glu^ neuronal activity may be associated with the depression condition in comorbidity mice.

### 2.2 The Increased PVPGlu Activity is Involved in the Painful Behavior in Comorbidity Mice

**Figure 2:**
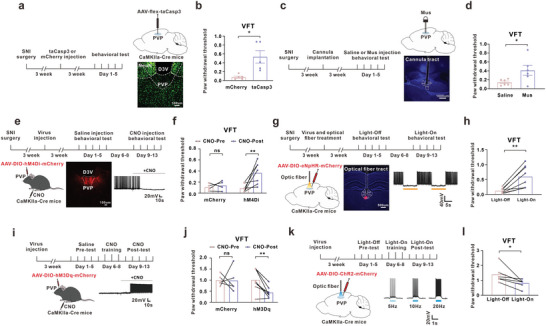
PVP^Glu^ hyperexcitability contributed to the painful behavior in the comorbidity mice. a) Schedule of experimental process (left) and schematic diagram (upper right) of the injection of AAV‐flex‐taCasp3 in PVP in CaMKIIa‐Cre mice. AAV‐flex‐taCasp3 application ablated the glutamatergic neurons of PVP in CaMKIIa‐Cre mice (lower right). Scale bar, 100 um. b) Ablation of PVP^Glu^ significantly attenuated the mechanical allodynia induced by SNI in CaMKIIa‐Cre mice (mCherry, n = 5 mice; taCasp3, n = 5 mice. t(8) = 3.191, p = 0.0128). c) Schedule of experimental process (left) and schematic diagram and typical image (right) for the injection location of the GABA_A_ receptor agonist muscimol (Mus). Scale bar, 1000 um. d) Intra‐PVP injection Mus relieved the mechanical allodynia in the comorbidity mice (Saline, n = 6 mice, Mus, n = 6 mice; t(10) = 2.321, p = 0.0427). e) Schedule and image for the injection of AAV‐DIO‐hM4Di‐mCherry into PVP and intraperitoneal injection CNO, and whole‐cell recording showed the inhibition of CNO on PVP^Glu^ neurons activity following intra‐PVP injection of AAV‐DIO‐hM4Di‐mCherry in CaMKIIa‐Cre mice. Scale bar, 100 um. f) The mechanical allodynia induced by SNI surgery was attenuated by intraperitoneal injection CNO following injection AAV‐DIO‐hM4Di‐mCherry into PVP in CaMKIIa‐Cre mice (mCherry, n = 4 mice; t(3) = 0.6441, p = 0.5654. hM4Di, n = 7 mice; t(6) = 3.383, p = 0.0148). g) Schedule and image for the injection of AAV‐DIO‐eNpHR‐mCherry into PVP, and yellow light inhibited the PVP^Glu^ neurons activity following intra‐PVP injection of AAV‐DIO‐eNpHR‐mCherry in CaMKIIa‐Cre mice. Scale bar, 500 um. h) The paw withdrawal threshold significantly increased by yellow light stimulation (594 nm, 10 mW, constant) in comorbidity mice after SNI surgery (eNpHR, n = 8 mice; t(7) = 4.373, p = 0.0033). i) Schedule and schematic diagram for the injection of virus, whole‐cell recording showed the activation of CNO on PVP^Glu^ neurons following intra‐PVP injection of AAV‐DIO‐hM3Dq‐mCherry in CaMKIIa‐Cre mice. j) Application with CNO (i.p.) decreased the paw withdrawal threshold in naïve CaMKIIa‐Cre mice with injection of AAV‐DIO‐hM3Dq‐mCherry (mCherry, n = 5 mice; t(4) = 0.0311, p = 0.9767. hM3Dq, n = 8 mice; t(7) = 4.258, p = 0.0038). k) Schedule and schematic diagram for the injection of optogenetic virus, and the traces to show that blue light activated the PVP^Glu^ neurons. l) The blue light stimulation (473 nm, 10 mW, 20 Hz, 5 ms) significantly induced painful behavior in naive mice (ChR2, n = 7 mice; t(6) = 3.35, p = 0.0154). All data were presented as the mean ± s.e.m. *P < 0.05, **P < 0.01. For detailed statistical information, see Supporting Table.

To identify the role of PVP^Glu^ neurons in comorbid behavior, we first ablated the glutamatergic neurons by injecting the AAV‐flex‐taCasp3 into the PVP of CaMKIIa‐Cre mice (**Figure** **2**a). The behavioral test revealed that ablation of PVP^Glu^ significantly alleviated the mechanical allodynia, but did not change the depression‐like behaviors (EPM, SPT, TST or FST) in comorbidity mice (Figure 2b; Figure S2a,b, Supporting Information). Microinfusion of the GABA_A_ receptor agonist Muscimol (MUS, 0.25 nmol, 150 nl) to rapidly inactivate PVP neurons attenuated mechanical allodynia in comorbidity mice (Figure 2c,d), while it did not affect the depression‐like behaviors (Figure S2c,d, Supporting Information). To further verify the role of PVP^Glu^ in comorbidity mice, we manipulated PVP^Glu^ activity using chemogenetic approach, in which the obvious mCherry‐positive signal in PVP indicated the effectiveness of virus transfection (Figure 2e). The results showed that application of CNO (clozapine‐N‐oxide, i.p.), which completely inhibited the PVP^Glu^ activity, reversed SNI‐induced mechanical allodynia, while it did not affect the depression‐like behavior in comorbidity mice (Figure 2e,f; Figure S2e,f, Supporting Information). The optogenetic results showed that yellow light stimulation (594 nm, 10 mW, constant) inhibited the activity of PVP^Glu^ neurons and attenuated the mechanical allodynia, but did not affect the depression‐like behaviors in CaMKIIa‐Cre mice with injection of AAV‐DIO‐eNpHR‐mCherry following SNI (Figure 2g,h; Figure S2g,h, Supporting Information). Moreover, in CaMKIIa‐Cre naive mice with injection of intra‐PVP AAV‐DIO‐hM3Dq‐mCherry (Figure 2i), CNO treatment increased the activity of PVP^Glu^ neurons and induced the mechanical allodynia (Figure 2i,j), but did not change depression‐like behavior (Figure S3a,b, Supporting Information). In addition, blue light (473 nm, 10 mW, 20 Hz, 5 ms) induced the hyperactivity of PVP^Glu^ neurons and mechanical allodynia (Figure 2k,l), but did not change the depression‐like behavior in CaMKIIa‐Cre naive mice with injection of AAV‐DIO‐ChR2‐mCherry into PVP (Figure S3c,d, Supporting Information). In addition, difference frequency of blue‐light stimulation and difference power of yellow‐light stimulation were clarified in painful behavior (Figure S4a–d, Supporting Information). The above results indicated that the increased PVP^Glu^ neuron activity contributed to the painful behavior in comorbidity mice.

### 2.3 The Decreased PVAGlu Activity is Involved in the Depression‐Like Behavior in Comorbidity Mice

**Figure 3:**
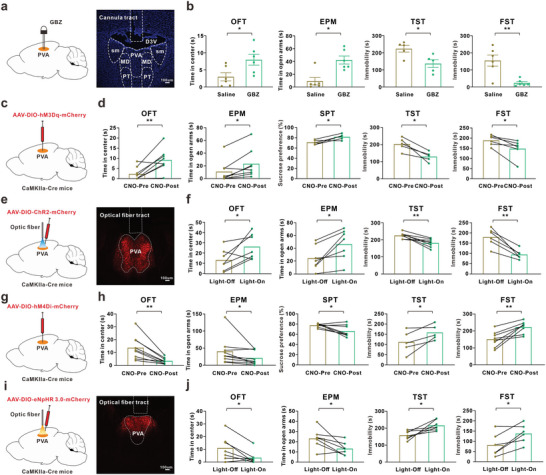
The reduced excitability of PVAGlu contributed to SNI‐induced depression‐like behaviors in mice. a) Schema of cannula implantation in PVA (left) and the representative image of cannula trace (right). Scale bar, 100 um. b) Microinjection of Gabazine (GBZ) into the PVA increased the time spent in center in OFT and in open arms in EPM, and also decreased the immobility time in TST and FST (OFT: Saline, n = 6 mice; GBZ, n = 6 mice; t(10) = 2.498, p = 0.0316. EPM: Saline, n = 6 mice; GBZ, n = 6 mice; U = 3, p = 0.0152. TST: Saline, n = 5 mice; GBZ, n = 5 mice; t(8) = 2.932, p = 0.0189. FST: Saline, n = 6 mice; GBZ, n = 6 mice; t(10) = 3.935, p = 0.0028). c) Scheme for the injection location of AAV‐DIO‐hM3Dq‐mCherry in PVA of CaMKIIa‐Cre mice. d) The depression‐like behaviors were explored before and after by intraperitoneal injection of CNO (OFT: hM3Dq, n = 8 mice; t(7) = 3.801, p = 0.007. EPM: hM3Dq, n = 8 mice; t(7) = 3.343, p = 0.012. SPT: hM3Dq, n = 5 mice; t(4) = 3.467, p = 0.026. TST: hM3Dq, n = 5 mice; t(4) = −4.117, p = 0.015. FST: hM3Dq, n = 6 mice; t(5) = −3.273, p = 0.022). e) Scheme of PVA injection with AAV‐DIO‐ChR2‐mCherry and the implantation of optical fiber for blue light stimulation (left) and the typical image of localized fluorescence and optical fiber trace (right). Scale bar, 100 um. f) Photostimulation attenuated the depression‐like behaviors in comorbidity mice after SNI treatment (OFT: ChR2, n = 7 mice; t(6) = 2.677, p = 0.037. EPM: ChR2, n = 7 mice; t(6) = 3.387, p = 0.015. TST: ChR2, n = 8 mice; t(7) = −4.567, p = 0.003. FST: ChR2, n = 6 mice; t(5) = −5.746, p = 0.002). g) Scheme for the injection location of AAV‐DIO‐hM4Di‐mCherry in PVA of CaMKIIa‐Cre mice. h) Intraperitoneal injection CNO significantly reduced the time spent in center of OFT, in open arms of EPM and the sucrose preference of SPT, and also increased the immobility time in TST and FST in naïve mice (OFT: hM4Di, n = 9 mice; t(8) = −4.361, p = 0.002. EPM: hM4Di, n = 9 mice; Z = −2.547, p = 0.011. SPT: hM4Di, n = 8 mice; t(7) = −2.796, p = 0.027. TST: hM4Di, n = 5 mice; t(4) = 2.953, p = 0.042. FST: hM4Di, n = 8 mice; t(7) = 4.367, p = 0.003). i) Scheme of PVA injection with AAV‐DIO‐eNpHR 3.0‐mCherry and the implantation of optical fiber for yellow light stimulation (left) and the typical image of localized fluorescence and optical fiber trace (right). Scale bar, 100 um. j) Stimulation with yellow light (594 nm) dramatically induced the depressive‐like behaviors in naïve mice (*t*‐test, *p < 0.05 versus the corresponding to before stimulation, eNpHR group (OFT: eNpHR, n = 7 mice; t(6) = −2.957, p = 0.025. EPM: eNpHR, n = 7 mice; t(6) = −2.816, p = 0.031. TST: eNpHR, n = 7 mice; t(6) = 3.321, p = 0.016. FST: eNpHR, n = 6 mice; t(5) = 3.336, p = 0.021). *P < 0.05, **P < 0.01. For detailed statistical information, see Supporting Table.

Next, we examined the role of the decreased activity of PVA^Glu^ in comorbidity mice after SNI treatment. Intra‐PVA injection of the GABA_A_ receptor antagonist Gabazine (GBZ), which increased the excitatory activity^[^ ^14^ ^]^ (**Figure** **3**a), effectively alleviated the depression‐like behavior, while it did not change the mechanical withdrawal threshold in the comorbidity mice (Figure 3b; Figure S5a,b, Supporting Information). In the CaMKIIa‐Cre mice with intra‐PVA injection of AAV‐DIO‐hM3Dq (Figure 3c), CNO treatment (i.p.) significantly improved the depression‐like behavior, but did not affect the mechanical allodynia in the comorbidity mice (Figure 3d; Figure S5c,d, Supporting Information). Similarly, activation of PVA^Glu^ neurons using the optogenetic method also increased the time spent in the center of OFT and the open arm of EPM, and decreased the immobility time in TST and FST, but did not change the mechanical withdrawal threshold in SNI mice (Figure 3e,f; Figure S5e,f, Supporting Information). Furthermore, inhibition of PVA^Glu^ neurons in naïve mice by using chemogenetics or optogenetics patterns induced the depression‐like behavior (Figure 3g,j), but did not induce mechanical allodynia (Figure S5g–j, Supporting Information). The results indicated that the reduced excitability of PVA^Glu^ neurons participated in the depression‐like behavior in comorbidity mice.

### 2.4 VLPAG^GABA^ is Involved in the Mechanical Allodynia in Comorbidity Mice

**Figure 4:**
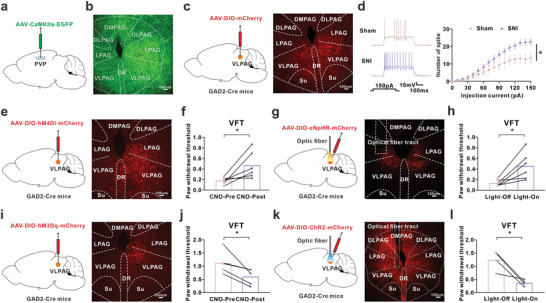
The enhanced excitability of VLPAG^GABA^ contributed to the chronic pain in the comorbid. a) Schema of PVP injection of AAV‐CaMKIIa‐EGFP in mice. b) Representative image of EGFP+ fiber in PAG of mice with PVP injection of AAV‐CaMKIIa‐EGFP. Scale bar, 100 µm. c) Schema of VLPAG injection AAV‐DIO‐mCherry in GAD2‐Cre mice (left) and typical images of restricted fluorescence in VLPAG (right). Scale bar, 100 µm. d) Representative traces (left) and summary (right) of action potential firing responsive to depolarizing current injections in VLPAG^GABA^ neurons (Sham, n = 15 cells from 3 mice; SNI, n = 23 cells from 4 mice; F(1, 36) = 5.42, p = 0.0256). e) Schema of VLPAG injection AAV‐DIO‐hM4Di‐mCherry in GAD2‐Cre mice and typical images of restricted fluorescence in VLPAG. Scale bar, 100 µm. f) The mechanical allodynia induced by SNI treatment was significantly alleviated by intraperitoneal application CNO (hM4Di, n = 6 mice; t(5) = 2.864, p = 0.035). g) Schema of injection of AAV‐DIO‐eNpHR‐mCherry into VLPAG and implantation of optic fiber in GAD2‐Cre mice (left) and the typical images of injection site (right). Scale bar, 100 µm. h) The paw withdrawal threshold was increased by inhibition of VLPAG^GABA^ with yellow light stimulation (eNpHR, n = 6 mice; t(5) = 3.375, p = 0.0198). i) Schema of VLPAG injection with AAV‐DIO‐hM3Dq‐mCherry in GAD2‐Cre mice and typical fluorescence images of injection site in VLPAG. Scale bar, 100 µm. j) Activation of VLPAG^GABA^ by CNO‐induced mechanical allodynia in the naïve mice (hM3Dq, n = 5 mice; t(4) = −3.276, p = 0.0306). k) Schema of injection of AAV‐DIO‐ChR2‐mCherry into VLPAG and implantation of optic fiber in GAD2‐Cre mice (left) and the typical images of injection site (right). Scale bar, 100 µm. l) The paw withdrawal threshold was decreased by activation of VLPAG^GABA^ with blue light stimulation in naïve mice (ChR2, n = 4 mice; t(3) = −3.952, p = 0.0289). All data were presented as the mean ± s.e.m. *P < 0.05, **P < 0.01. For detailed statistical information, see Supplementary Table.

To elucidate the neuronal circuit for the PVP^Glu^ neurons to regulate mechanical allodynia, we first infused AAV‐CaMKIIa‐EGFP into PVP. Three weeks later, the fluorescence expression of EGFP+ fibers was observed in many regions, including PAG, central amygdala (CeA), prelimbic (PrL), and zona incerta (ZI) (**Figure** **4**a,b; Figure S6a,b, Supporting Information). It is well known that the PAG is an evolutionarily conserved region in the midbrain and compose of DMPAG, DLPAG, LPAG, and VLPAG.^[^ ^15^ ^]^ Studies showed that VLPAG GABAergic neurons (VLPAG^GABA^) was involved in the regulation of nocifensive behaviors following nerve injury or inflammatory responses.^[^ ^16^ ^]^ To confirm whether VLPAG^GABA^ contributes to chronic pain in the comorbid, we first examined the excitability of VLPAG^GABA^. The results showed that the number of action potential of VLPAG^GABA^ (mCherry‐positive cells) was higher in comorbidity mice than that in the sham mice (Figure 4c,d). Moreover, we injected the Cre‐dependent chemogenetic and optogenetic virus (AAV‐DIO‐hM4Di/eNpHR‐mCherry) into VLPAG of GAD2‐cre mice (Figure 4e,g). Inhibition of VLPAG^GABA^ with CNO or yellow‐light treatment significantly increased the paw withdrawal threshold in SNI‐treated comorbidity mice (Figure 4f), whereas activation of VLPAG^GABA^ with CNO or blue‐light treatment dramatically decreased the paw withdrawal threshold in naïve mice (Figure 4i–l). These results suggested that the increased excitability of VLPAG^GABA^ contributed to the mechanical allodynia in the comorbidity mice.

### 2.5 The PVPGlu →VLPAGGABA Circuit Contributed to the Mechanical Allodynia in Comorbidity Mice

**Figure 5:**
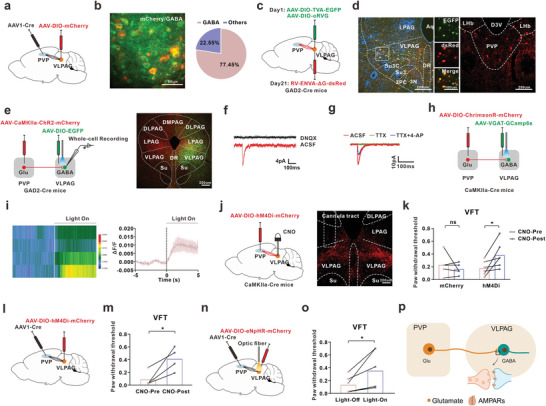
The PVP^Glu^→VLPAG^GABA^ circuit contributed to the mechanical allodynia in comorbidity mice. a) Schema of virus injection in mice. b) Representative image and quantitative analysis of the colocalization of mCherry‐positive neurons and GABA‐positive in VLPAG. Scale bar, 50 µm (n  =  25 images from 3 mice). c) Schema of the Cre‐dependent retrograde trans‐monosynaptic RV tracing strategy in GAD2‐Cre mice. d) Left: typical image of the injection site and viral expression within the VLPAG of GAD2‐Cre mice. Starter cells (yellow) co‐express AAV‐DIO‐TVA‐EGFP, AAV‐DIO‐RVG (green) and RV‐ENVA‐ΔG‐DsRed (red). Scale bar, 200 µm. The middle images depicted the area shown in the dotted line square of the VLPAG. Scale bar, 100 µm. Right: DsRed‐labeled neurons within the PVP. Scale bar, 200 µm. e) Schema of PVP injection of AAV‐CaMKIIa‐ChR2‐mCherry and VLPAG injection of AAV‐DIO‐EGFP in GAD2‐Cre mice and the recording configuration in acute slices (Left). Typical images of the injection site and viral expression (Right). Scale bar, 200 µm. f) Representative traces for the recording of light‐evoked current (473 nm, 20 ms) before and after DNQX (20 µM) treatment. g) One example of EPSCs recorded in VLPAG^GABA^ neuron in ACSF and after the sequential application of TTX (1 µM) and 4‐AP (100 µM). h) Schema of virus injection and optical fiber photometer recording configuration. i) The heatmaps (left) and the mean (right) showed that the Ca^2+^ signal rapidly increased by the persistent red‐light stimulation (635 nm) in CaMKIIa‐Cre mice with PVP injection of AAV‐DIO‐ChrimsionR‐mCherry and VLPAG injection of AAV‐CaMKIIa‐GCamp6s (n = 4). j) Schema of injection of AAV‐DIO‐hM4Di‐mCherry into PVP of CaMKIIa‐Cre mice and implantation of cannula into VLPAG (Left) and typical images of mCherry+ fiber and cannula tract (Right). Scale bar, 200 µm. k) Intra‐VLPAG injection of CNO relieved the SNI‐induced mechanical allodynia in the mice microinjected with AAV‐DIO‐hM4Di‐mCherry into PVP of CaMKIIa‐Cre mice (mCherry, n = 5 mice; Z = −0.365, p = 0.715. hM4Di, n = 6 mice; Z = −2.201, p = 0.028). l) Schema of injection of AAV1‐hSyn‐Cre into PVP and AAV‐DIO‐hM4Di‐mCherry into VLPAG. m) The mechanical allodynia was rescued by intraperitoneal injection of CNO in SNI‐treated comorbidity mice (hM4Di, n = 4 mice; t(3) = 3.481, p = 0.04). n) Schema of injection of AAV1‐hSyn‐Cre into PVP and AAV‐DIO‐eNpHR‐mCherry into VLPAG and implantation of cannula into VLPAG. o) The paw withdrawal threshold was increased through inhibition PVP‐projected VLPAG neurons with light stimulation (eNpHR, n = 6 mice; t(5) = 2.802, p = 0.038). p) Schema of PVP^Glu^ projections onto VLPAG^GABA^ neurons. The schematic diagram was created with BioRender.com. *P < 0.05. For detailed statistical information, see Supporting Table.

We further identified whether the PVP^Glu^ projected to VLPAG^GABA^ and mediated the chronic pain in the SNI‐induced comorbidity mice. Following intra‐PVP injection of AAV1‐hSyn‐Cre and intra‐VLPAG injection of AAV‐DIO‐mCherry, the double immunofluorescence assay revealed that 77.45% of mCherry‐positive cells were co‐localized with the GABA‐specific immunosignal (**Figure** **5**a,b). Furthermore, we used a specific retrograde trans‐monosynaptic tracing system involving the VLPAG infusion of Cre‐dependent helper viruses and RV in the GAD2‐Cre mice (Figure 5c,d, left and middle) to confirm the projection from PVP^Glu^ to VLPAG^GABA^. We identified the intensively DsRed‐labelled neurons in the PVP, CeA, IPAC, LH, mPFC, and ZI (Figure 5d, right; Figure S6c,d, Supporting Information). These results suggested that PVP^Glu^ neurons preferentially projected onto VLPAG^GABA^ neurons. To identify the function of PVP^Glu^→VLPAG^GABA^ circuits, optogenetics experiments were first performed (Figure 5e). Whole‐cell recording in brain slices showed that blue light stimulation (473 nm; 5 ms) of ChR2‐containing PVP^Glu^ terminals in VLPAG induced excitatory postsynaptic currents (EPSCs), which were blocked by the AMPAR antagonist DNQX (20 µM) (Figure 5f). The EPSCs were eliminated after bath application of tetrodotoxin (TTX, 1 µM) but then reintroduced by a 4‐aminopyridine (4‐AP, 100 µM) bath application (Figure 5g). In addition, the results of optical fiber photometer recordings showed that activation of PVP^Glu^ terminals increased the Ca^2+^ signals in VLPAG^GABA^ neurons (Figure 5h,i). These data demonstrated the monosynaptic connection between the PVP^Glu^ neurons and VLPAG^GABA^ neurons. Importantly, we injected AAV‐DIO‐hM4Di‐mCherry into PVP in the CaMKIIa‐Cre mice and found that inhibition of the PVP^Glu^ projection terminal in the VLPAG region by CNO microinjection significantly alleviated the mechanical allodynia (Figure 5j,k). Blocking of PVP^Glu^–projected VLPAG^GABA^ activity by the chemogenetic methods attenuated the mechanical allodynia in comorbidity mice (Figure 5l,m). Similarly, inhibition of PVP^Glu^–projected VLPAG^GABA^ activity by the optogenetics methods also alleviated the mechanical allodynia in the comorbidity mice (Figure 5n,o). Moreover, activation of the PVP^Glu^ projection terminal in the VLPAG region by CNO microinjection induced the mechanical allodynia in naïve (Figure S7a,b, Supporting Information), and PVP^Glu^–projected VLPAG^GABA^ hyperexcitability by using chemogenetic and optogenetic methods decreased the mechanical withdrawal threshold in naïve mice (Figure S7c–f, Supporting Information). These results suggested that the PVP^Glu^→VLPAG^GABA^ circuit participated in the painful behavior in comorbid (Figure 5p).

### 2.6 NAc^D2^ is Involved in the Depression‐Like Behavior in Comorbidity Mice

**Figure 6:**
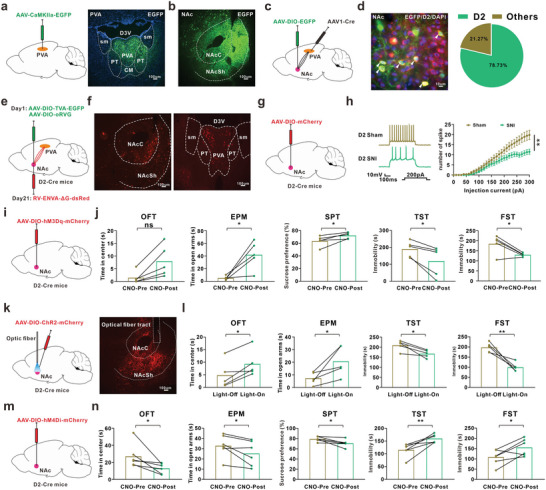
NAc^D2^ participated in the depression‐like behavior in comorbidity mice. a) Schema of PVA injection of AAV‐CaMKIIa‐EGFP in mice (Left) and typical image of injection site (Right). Scale bar, 100 µm. b) Representative image of EGFP^+^ fiber in NAc of mice with PVA injection of AAV‐CaMKIIa‐EGFP. Scale bar, 100 µm. c) Schematic of virus injection. d) Images (left) and statistic pie chart (right) showing that EGFP‐labeled neurons were colocalized with D2‐positive neurons in NAc. Scale bar, 10 µm (n  =  16 images from 3 mice). e) Schema of the Cre‐dependent retrograde trans‐monosynaptic RV tracing strategy in D2‐Cre mice. f) Left: typical image of the injection site and viral expression in the NAc of D2‐Cre mice. Scale bar, 100 µm. Right: DsRed‐labeled neurons within the PVA. Scale bar, 100 µm. g) Schema of NAc injection of AAV‐DIO‐mCherry in D2‐Cre mice. h) Representative traces and summary (right) of action potential firing rates in response to depolarizing current injections in D2 neurons (Sham, n = 21 neurons from 4 mice; SNI, n = 26 neurons from 4 mice; F(1, 45) = 6.812, p = 0.0122). i) Schema of injection of AAV‐DIO‐hM3Dq‐mCherry into NAc. j) The depression‐like behaviors were explored before and after by intraperitoneal injection of CNO (OFT: hM3Dq, n = 5 mice; t(4) = 3.319, p = 0.029. EPM: hM3Dq, n = 5 mice; t(4) = 00617, p = 0.022. SPT: hM3Dq, n = 5 mice; t(4) = 3.167, p = 0.034. TST: hM3Dq, n = 5 mice; t(4) = −3.407, p = 0.027. FST: hM3Dq, n = 5 mice; t(4) = −3.169, p = 0.034). k) Schema of injection of AAV‐DIO‐ChR2‐mCherry and implantation of optical fiber into NAc (left), and typical image of virus expression and implantation site in NAc in D2‐Cre mice (Right). Scale bar, 100 µm. l) The depression‐like behaviors were examined before and after blue light stimulation (OFT: ChR2, n = 5 mice; t(4) = 3.913, p = 0.017. EPM: ChR2, n = 5 mice; t(4) = 2.939, p = 0.042. TST: ChR2, n = 5 mice; t(4) = −3.852, p = 0.018. FST: ChR2, n = 5 mice; t(4) = −5.177, p = 0.007). m) Schema of NAc injection of AAV‐DIO‐hM4Di‐mCherry in D2‐Cre mice. n. Continuous injection of CNO induced depression‐like behaviors. (OFT: hM3Dq, n = 6 mice; t(5) = −2.614, p = 0.047. EPM: hM3Dq, n = 6 mice; t(5) = −2.629, p = 0.047. SPT: hM3Dq, n = 6 mice; t(5) = −2.971, p = 0.031. TST: hM3Dq, n = 6 mice; t(5) = 4.242, p = 0.008. FST: hM3Dq, n = 6 mice; t(5) = 2.849, p = 0.036). *P < 0.05, **P < 0.01. For detailed statistical information, see Supporting Table.

Evidence showed that NAc was composed of dopamine receptor 1‐expresing (D1) cells and dopamine receptor 2‐expresing (D2) cells,^[^ ^17^ ^]^ and it exhibited a key function in the emotional disorder behavior. To identify whether PVA^Glu^ neurons can project to the NAc to participate in the depression‐like behavior in comorbidity mice, we first dissected the fine PVA^Glu^ **→**NAc circuit. Following intra‐PVA injection of AAV‐CaMKIIa‐EGFP (**Figure** **6**a), multiple brain regions including NAc, AOM, LaVM, MnR, and VP showed obvious green fluorescence signals of EGFP ^+^ fiber (Figure 6b; Figure , Supporting Information). Furthermore, by injecting the anterograde trans‐monosynaptic virus (AAV1‐hSyn‐Cre) into the PVA and AAV‐DIO‐EGFP into NAc, the double immunostaining revealed that 78.73% EGFP‐positive cells were overlapped with D2‐specific immunosignal in the NAc (Figure 6c,d), suggesting that PVA^Glu^ neurons preferentially projected onto NAc D2 neurons (NAc^D2^).

Moreover, using a specific retrograde trans‐monosynaptic tracing system involving the NAc infusion of Cre‐dependent helper viruses and RV in the D2‐Cre mice (Figure 6e), we confirmed that, besides LO, PrL, AHP, and MD, PVA also sent fiber projections to the NAc^D2^ (Figure 6f; Figure S8c,d, Supporting Information). Next, we observed the excitability of NAc^D2^ by labeling the D2‐positive neurons with AAV‐DIO‐mCherry in D2‐Cre mice with electrophysiological and Ca^2+^ signal assays. We found that the number of evoked action potential and Ca^2+^ signal of D2‐positive neurons significantly decreased in comorbidity mice (Figure 6g,h; Figure S8e,f, Supporting Information). Importantly, behavioral test showed that activation of D2‐positive neurons with CNO or blue‐light stimulation significantly alleviated the depression‐like behavior of comorbidity mice (Figure 6i–l). Conversely, continuous inhibition of D2‐positive neurons with CNO induced the depression‐like behavior in naïve mice (Figure 6m,n; Figure S8g, Supporting Information). Taken together, these results suggested that activation of the NAc^D2^ contributed to the depression‐like behavior in comorbid mice induced by SNI.

### 2.7 Inhibition of the PVA^Glu^→NAc^D1^ *^→^* ^D2^ Circuit Contributed to the Depression‐Like Behavior in Comorbidity Mice

**Figure 7:**
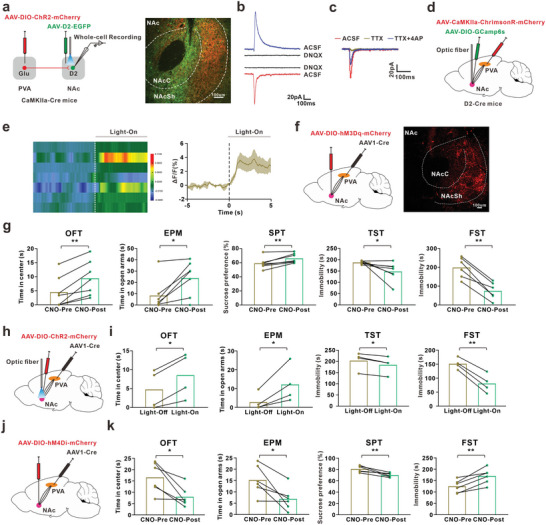
The role of PVA^Glu^→NAc^D2^ in the depression‐like behavior in comorbidity mice. a) Schema of PVA injection of AAV‐DIO–ChR2–mCherry and NAc injection of AAV‐D2–EGFP in CaMKII‐Cre mice (Left) and typical images of injection site and viral expression in NAc (Right). Scale bar, 100 µm. b) Blue light stimulation (470 nm, 5 ms) of PVA^Glu^ fiber induced an EPSCs and an IPSCs in each responsive NAc^D2^ neurons, and blocked by bath application of DNQX (20 µM). c) An example of EPSCs recorded in NAc^D2^ neuron in ACSF and after the sequential application of TTX (1 µM) and 4‐AP (100 µM). d) Schematic of PVA injection of AAV‐CaMKIIa‐ChrimsonR‐mCherry and NAc injection of AAV‐DIO‐GCamp6s in D2‐Cre mice. e) The heatmaps (left) and the mean (right) show that Ca2+ signal rapidly increased with persistent red‐light stimulation (635 nm) in D2‐Cre mice with PVA injection of AAV‐CaMKIIa‐ChrimsionR‐mCherry and NAc injection of AAV‐DIO‐GCamp6s (n = 7). f) Schema of injection of AAV1‐hSyn‐Cre into PVA and AAV‐DIO‐hM3Dq‐mCherry into NAc (left), and typical image of injection site in NAc (Right). Scale bar, 100 µm. g) The depression‐like behaviors were explored before and after intraperitoneal injection of CNO (OFT: hM3Dq, n = 7 mice; t(6) = 4.89, p = 0.003. EPM: hM3Dq, n = 7 mice; t(6) = 3.195, p = 0.019. SPT: hM3Dq, n = 7 mice; t(6) = 4.152, p = 0.006. TST: hM3Dq, n = 7 mice; t(6) = −2.452, p = 0.0497. FST: hM3Dq, n = 6 mice; t(5) = −7.865, p = 0.001). h) Schema of injection of AAV1‐hSyn‐Cre into PVA and AAV‐DIO‐ChR2‐mCherry into NAc, and implantation of optical fiber into NAc. i) The depression‐like behaviors were tested before and after blue‐like stimulation (OFT: ChR2, n = 4 mice; t(3) = 5.744, p = 0.0105. EPM: ChR2, n = 4 mice; t(3) = 3.419, p = 0.042. TST: ChR2, n = 4 mice; t(3) = −5.665, p = 0.011. FST: ChR2, n = 4 mice; t(3) = −10.205, p = 0.002). j) Schema of PVA injection of AAV1‐Cre and NAc injection of AAV‐DIO‐hM4Di‐mCherry. k) Depression‐like behaviors were performed before and after injection of CNO (OFT: hM3Dq, n = 6 mice; t(5) = −3.512, p = 0.017. EPM: hM3Dq, n = 6 mice; t(5) = −3.233, p = 0.023. SPT: hM3Dq, n = 6 mice; t(5) = −4.036, p = 0.01. FST: hM3Dq, n = 6 mice; t(5) = 5.382, p = 0.003). *P < 0.05, **P < 0.01. For detailed statistical information, see Supporting Table.

To identify whether PVA^Glu^ **→**NAc^D2^ circuit participated in the depression‐like behavior in comorbidity mice, we first examined the functional connection of PVA^Glu^ **→**NAc^D2^ circuit by intra‐PVA injection of AAV‐DIO‐ChR2‐mCherry and intra‐NAc injection of AAV‐D2‐EGFP in CaMKIIa‐Cre mice (**Figure** **7**a). Whole‐cell recording revealed that blue‐light stimulation of ChR2‐containing PVA^Glu^ terminals in NAc^D2^ induced excitatory postsynaptic currents (EPSCs), which were blocked by the AMPAR antagonist DNQX (20 µM) (Figure 7b). The EPSCs were eliminated after bath application of TTX (1 µM) but then reintroduced by 4‐AP (100 µM) bath application (Figure 7c). Meanwhile, the increased calcium signals in the PVA^Glu^ terminals in NAc^D2^ also confirmed the functional connectivity (Figure 7d,e), further demonstrating the monosynaptic connection between the PVA^Glu^ neurons and NAc^D2^ neurons.

Next, we microinjected AAV1‐hSyn‐Cre into PVA and Cre‐dependent chemogenetics and optogenetics viruses into NAc to manipulate NAc neurons receiving PVA projection. Activation of PVA‐projected NAc neurons with intraperitoneal injection of CNO or blue‐light stimulation (473 nm) respectively attenuated depression‐like behaviors in comorbidity mice (Figure 7f–i). Consistently, inhibition of PVA‐projected NAc neurons with continuous infusion of CNO induced depression‐like behavior in naïve mice (Figure 7j,k). These results showed that the PVA^Glu^ **→**NAc^D2^ circuit participated in the depression‐like behavior in comorbidity mice.

**Figure 8:**
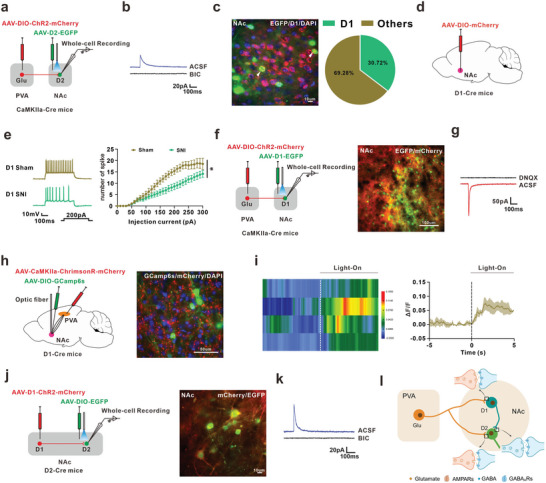
The local NAc^D1→D2^ circuits. a) Schema of PVA injection of AAV‐DIO–ChR2–mCherry and NAc injection of AAV‐D2–EGFP in CaMKII‐Cre mice. b) The IPSCs were blocked by the incubation of bicuculline (BIC, 10 µM). c) Images (left) and statistic pie chart (right) showed that EGFP‐labeled neurons were colocalized with D2‐positive neurons in NAc. Scale bar, 10 µm (n  =  14 images from 3 mice). d) Schema of NAc injection of AAV‐DIO‐mCherry in D1‐Cre mice. e) Representative traces (left) and summary (right) of action potential firing rates in response to depolarizing current injections in D1 neurons (Sham, n = 18 cells from 4 mice; SNI, n = 21 cells from 4 mice; F(1, 37) = 7.155, p = 0.0111). f) Schema of PVA injection of AAV‐DIO–ChR2–mCherry and NAc injection of AAV‐D1–EGFP in CaMKII‐Cre mice and the recording configuration in the acute brain slices (left), and typical image of virus expression (right). Scale bar. 100 µm. g) Optostimulation (blue light, 470 nm, 5 ms) of PVA^Glu^ fiber terminal induced an EPSCs in NAc^D1^ neurons, which were blocked by the bath application of DNQX (20 µM). h) Schema of PVA injection of AAV‐CaMKIIa‐ChrimsonR‐mCherry and NAc injection of AAV‐DIO‐GCamp6s in D1‐Cre mice (left), and typical image of viral expression of injection site (right). Scale bar, 50 µm. i) The heatmaps (left) and the mean (right) showed that Ca^2+^ signal rapidly increased following persistent red‐light stimulation (635 nm) in D1‐Cre mice (n = 4). j) Schema of injection of AAV‐D1‐ChR2‐mCherry and AAV‐DIO‐EGFP into NAc in D2‐Cre mice (left), and typical images of injection site and viral expression in NAc (right). Scale bar, 10 µm. k) For recording in the NAc^D2^ neurons, optostimulation (blue light, 470 nm, 5 ms) induced an IPSC, which was blocked by incubation of BIC (10 µM). l) A model of long‐range and local PVA^Glu^→ NAc^D1→D2^ circuits. The schematic diagram was created with BioRender.com. *P < 0.05. For detailed statistical information, see Supporting Table.

Interestingly, in addition to EPSCs, inhibitory postsynaptic currents (IPSCs) were also recorded in NAc^D2^ with ChR2‐containing PVA^Glu^ projections, which was prevented by GABA_A_R antagonist bicuculline (BIC, 20 µM) (**Figure** **8**a,b). This raised the possibility that the PVA^Glu^ projected to another primary type of neurons, NAc^D1^ neurons, which subsequently innervated to local NAc^D2^ neurons. Therefore, we first observed whether PVA^Glu^ projected to NAc^D1^ neurons. The double immunostaining revealed that 30.72% EGFP‐positive cells overlapped with D1‐specific immunosignal in the NAc (Figure 6c and Figure 8c). The excitability of NAc^D1^ neurons was examined by labeling the D1 neurons with AAV‐DIO‐mCherry in D1‐Cre mice, and the number of action potential of NAc^D1^ in comorbidity mice was lower than that in the sham mice (Figure 8d,e). To investigate the potential connection of PVA^Glu^ **→**NAc^D1^ circuit, we injected AAV‐DIO‐ChR2‐mCherry into PVA and AAV‐D1‐EGFP into NAc in CaMKIIa‐Cre mice (Figure 8f). Whole‐cell recording results showed that blue‐light stimulation of ChR2‐containing PVA^Glu^ terminals in NAc^D1^ induced the EPSCs, which was blocked by DNQX (Figure 8g). In addition, the calcium signals of NAc^D1^ neurons were increased by red‐light stimulation of ChrimsonR‐containing PVA^Glu^ terminals in NAc^D1^ neurons (Figure 8h,i). To identify whether PVA^Glu^ **→**NAc^D1^ circuit participated in the depression‐like behavior in comorbidity mice, we microinjected AAV1‐hSyn‐Cre into PVA and AAV‐D1‐DIO‐hM3Dq‐mCherry into NAc to manipulate NAc^D1^ neurons receiving PVA projection (Figure S9a, Supporting Information). Activation of PVA‐projected NAc^D1^ neurons with intraperitoneal injection of CNO did not change the depression‐like behavior in comorbid mice (Figure S9b, Supporting Information). Consistently, inhibition of PVA‐projected NAc^D1^ neurons with continuous infusion of CNO did not induce depression‐like behavior in naïve mice (Figure S9c, Supporting Information). These results suggested that PVA regulates depression‐like behavior in comorbidity mice with chronic pain and depression through NAc^D2^ neurons, rather than NAc^D1^ neurons. Notably, following infusion of AAV‐D1‐ChR2‐mCherry and AAV‐DIO‐EGFP into NAc of D2‐Cre mice, the whole‐cell recording showed that blue‐light stimulation evoked IPSCs in NAc^D2^ neurons, which were blocked by BIC (10 µM) (Figure 8j,k). Taken together, these data confirmed the functional PVA^Glu^→NAc^D1→D2^ circuits participated in depression‐like behavior in comorbidity mice (Figure 8l).

## 3 Discussion

Evidence indicates that chronic pain and depression‐like behavior often coexist in multiplied diseases. The paraventricular thalamus (PVT) is a midline thalamic brain region that has emerged as a critical circuit node to modulate positive and negative experiences and behaviors. However, how the PVT regulates the chronic painful behavior and depression‐like behavior in the comorbidity setting remains unclear. Here, we found that the anterior (PVA) and posterior (PVP) subregions of paraventricular thalamus exhibited distinctive activity in chronic pain/depression comorbidity mice. Furthermore, the results of loss‐ or gain‐of‐function revealed that the enhanced activity of PVP glutamatergic neurons (PVP^Glu^) participated in the chronic painful behavior, while the decreased PVA glutamatergic neurons (PVA^Glu^) activity was involved in depression‐like behavior in comorbidity mice, respectively. The results of viral tracing and electrophysiology showed that PVP^Glu^ neurons had the functional projection to the ventrolateral periaqueductal gray GABAergic neurons (VLPAG^GABA^), and PVA^Glu^ can functionally project to nucleus accumbens D2‐positive neurons (NAc^D2^) with a local circuit from D1‐positive neurons to D2‐positive neurons. Activation and inhibition of neuronal activity by chemogenetic and optogenetic assays revealed that the circuit of PVP^Glu^ →VLPAG^GABA^ was responsible for chronic painful behavior and PVA^Glu^ →NAc^D1^ *^→^* ^D2^ contributed to the depression‐like behavior induced by SNI (Figure S9, Supporting Information). Our findings suggested that different neural circuits originating from the PVT may mediate the formation of chronic pain and depression, which ultimately leading to the occurrence of comorbidity.

Thalamus is located in the center core of brain and forms extensive connections with other brain regions, and involved in processing sensory information, emotions regulation, and formation and storage of memories.^[^ ^4^, ^18^ ^]^ PVT, as one of the important nuclei in the thalamus, is mainly composed of glutamatergic neurons and plays a critical role in the motivated behavior.^[^ ^5^, ^19^ ^]^ Here, we first observed that SNI increased the cFos expression in PVP, but not PVA. Electrophysiological results showed that the number of evoked action potential of the labeled PVP^Glu^ obviously increased in SNI‐induced comorbidity mice. Furthermore, the calcium signals of PVP^Glu^ were both significantly increased in the comorbidity setting or mechanical stimuli. While some studies showed that PVA might be involved in neuropathic pain,^[^ ^20^ ^]^ the present study showed that ablation or inhibition of PVP^Glu^ attenuated mechanical allodynia in comorbidity mice, though it did not have a substantial effect on the depression‐like behavior. Moreover, activation of PVP^Glu^ by using chemogenetic or optogenetic methods induced the mechanical allodynia, but not depression‐like behavior, in naïve mice. Similar studies have shown that lesioning PVP neurons can slightly relieve mechanical allodynia within 10 days following SNI.^[^ ^21^ ^]^ Clinical and preclinical investigations have shown that multiple factors including environmental and psychological aspects can bring out the adaptive modifications of brain structure and brain function to lead to chronic pain.^[^ ^22^ ^]^ It is possible that nerve injury induces the synaptic and functional reassembly of PVP to mediate the chronic painful behavior in comorbidity. Interestingly, ablation of PVP^Glu^ neurons did not impact on the depression‐like behavior but increased time in center area of open field test. After detailed examination of the range of neuronal ablation in the intervention mice, we have a few hypotheses, one of which may be related to functional compensation, the other may be related to the complexity of anxiety comorbidity, and the third may be related to the functional complexity of the PVP brain region. Although the PVT has been acknowledged to be an important node in the emotional processing network for decades,^[^ ^10^ ^]^ extremely limited evidence provides few clues for the potential role of the PVT in depression‐like behavior. For PVA, evidence showed that PVA was involved in the behavior of feeding, rewarding, and anxiety.^[^ ^23^ ^]^ Here, the results of electrophysiology and the calcium signals indicated that the activity of PVA^Glu^ was significantly decreased in the comorbidity mice following SNI treatment, and the stimulation of FST or TST, but not mechanical stimuli, significantly reduced the Ca^2+^ signal in PVA^Glu^ in naive mice and comorbid mice. Moreover, activation of PVA^Glu^ by GBZ, chemogenetics or optogenetics alleviated the depression‐like behavior in the comorbidity mice, and inhibition of PVA^Glu^ induced the depression‐like behavior in naïve mice. Importantly, manipulating PVA^Glu^ activity did not alter painful behavior in comorbidity or naïve mice. These results first indicated that PVT participated in somatosensory (painful behavior) and affective dimensions (depression‐like behavior) of neuropathic pain may originate from different subdivisions (PVP and PVA) of this brain region.

It is reported that PVT projects to multiple brain regions including the central amygdala, mPFC BNST, NAc, respectively, by which it participates in arousal, conditioned fear, anxiety, and drug‐seeking.^[^ ^10^ ^]^ Evidence showed that PVP^Glu^, as the primary cell type in PVP, exerted the function by projection on several distal brain regions such as prelimbic cortex (PrL), the central nucleus of the amygdala (CeA) and NAc.^[^ ^24^ ^]^ Here, combining the virus trace, the Ca^2+^ signal assay and electrophysiology, we identified an excitability projection from the PVP^Glu^ to VLPAG^GABA^ (PVP^Glu^→VLPAG^GABA^). Inhibition of PVP^Glu^→VLPAG^GABA^ by chemogenetic and optogenetic methods prevented the mechanical allodynia, and activation of PVP^Glu^→VLPAG^GABA^ decreased the mechanical withdrawal threshold in naïve mice. Besides the PVP^Glu^ neurons, the VLPAG^GABA^ also received the projections from LHb, CeA, LH, mPFC, ZI and so on. Studies show that mPFC, NAc, and CeA projecting to VLPAG^GABA^ were involved in the pain hypersensitivity.^[^ ^25^ ^]^ The present data suggested that the circuit of PVP^Glu^→VLPAG^GABA^ mediated the painful behavior in the setting of comorbidity induced by nerve injury.

NAc comprises D1 receptor‐positive and D2 receptor‐positive neurons and plays an important role in the rewarding effect and emotion.^[^ ^26^ ^]^ Our data revealed that the activity of NAc D2 receptor‐positive neurons (NAc^D2^) was significantly decreased in comorbidity mice. Importantly, behavioral tests showed that activation of NAc^D2^ with CNO or blue‐light stimulation significantly alleviated the depression‐like behavior in comorbidity mice, and continuous inhibition of NAc^D2^ induced the depression‐like behavior in naïve mice. In the present study, we further found that PVA^Glu^ neurons preferentially projected onto NAc^D2^ neurons, compared with NAc^D1^ neurons. In fact, besides PVA^Glu^ neurons, PVP^Glu^ neurons also project to NAc, though difference exists. Studies have shown that PVA projects preferentially to the dorsomedial side of NAc, while PVP projects more to the ventromedial side of NAc.^[^ ^9b^ ^]^ The study showed that the activation of NAcSh‐projecting VTA DA terminals alleviated the CFA‐induced anhedonia‐like behavior without altering CFA‐induced hyperalgesia.^[^ ^27^ ^]^ Here, we found that the activation of PVA‐projected NAc neurons with intraperitoneal injection of CNO or blue‐light stimulation attenuated depression‐like behaviors in comorbidity mice, and inhibition of PVA‐projected NAc neurons induced depression‐like symptoms in naïve mice. However, activation or inhibition of PVA‐projected NAc^D1^ neurons did not change the depression‐like behavior. These results suggested that PVA^Glu^ **→**NAc^D2^ was involved in the depression‐like behavior in the comorbidity mice. Previous studies shown that both D1‐positive and D2‐positive neurons might be involved in depression‐like behavior.^[^ ^28^ ^]^ However, manipulation of PVA‐projected NAc^D1^ neurons did not show the impact on the depression‐like behavior. This raised the possibility that NAc^D1^ neurons might not regulate depression‐like behavior in comorbid mice, or that NAc^D1^ neurons only received limited PVA projection leading to insufficient drive for the regulation of depression‐like behavior. Interestingly, whole‐cell recording of D2‐positive neurons revealed that blue‐light stimulation of ChR2‐containing PVA^Glu^ terminals in NAc induced both excitatory postsynaptic currents (EPSCs) and inhibitory postsynaptic currents (IPSCs), and further analysis defined a microcircuit organization involving local NAc^D1^ neurons projecting inhibitory afferents with NAc^D2^ neurons. Previous report existed to show the reciprocal projecting microcircuit between NAc^D2^ and NAc^D1^ neurons.^[^ ^29^ ^]^ It is well known that the NAc^D1^ is an inhibitory GABAergic neuron, hence the decreased activity of D1‐positive neurons could tentatively disinhibit the D2‐positive neurons activity. However, we observed the decreased excitability in both D1‐positive and D2‐positive neurons in comorbidity mice, which likely resulted from the observation in the present study that PVA^Glu^ had a predominant projection on D2‐positive neurons relative to D1‐positive neurons. While manipulating NAc^D1^ neurons receiving PVA projection does not change depression‐like behavior, NAc^D1^ neurons have the ability to control NAc^D2^ neurons' excitability. Taken together, our results dissected the functional organization of PVA^Glu^→NAc^D1→D2^ circuits, which was involved in the depression‐like behavior in comorbidity mice.

There are still some unelucidated mechanisms in this study. Given that PVP can project to PrL, CeA, ZI, and other brain regions, it is noteworthy to further investigate whether these neural circuits are also involved in the regulation of mechanical allodynia in comorbid mice. Furthermore, PVA has the projections to various brain regions, including AOM, LaVM, MnR, and VP, which necessitates the further study to clarify the role of these neural circuits in the regulation of depression‐like behavior in comorbid mice.

In summary, our study dissected two discrete PVT‐originating neural circuits. In the rodent model of SNI, PVP^Glu^→VLPAG^GABA^ circuit was activated to mediate the painful behavior of comorbidity symptoms induced by nerve injury. Meanwhile, the excitability of PVA^Glu^→NAc^D1→D2^ circuit was decreased to participate in the depression‐like behavior in comorbidity mice. These two pathways synergistically contributed to the formation of comorbidity in the setting of nerve injury, which may provide potential insights for developing the optimal treatments for chronic pain and depression comorbidity.

## 4 Experimental Section

### Animals

In the present study, male mice (C57BL/6J) with age of more than 8 weeks were purchased from the Institute of Experimental Animals of Sun Yat‐sen University. The CaMKIIa‐Cre, GAD2‐Cre mice, D1‐Cre mice, and D2‐Cre mice were graciously provided by the colleagues in Southern Medical University. The genotype of all mice were verified. The animals were housed on a 12‐h light‐dark cycle (7:00 a.m. to 7:00 p. m.) with free access to food and water in a stable environment (temperature, 23 – 25 °C, humidity 30% – 70%), and all behavioral experiments were performed during the light cycle. All experimental protocols were approved by the Institutional Animal Care Committee and carried out in accordance with the National Institutes of Health Guide for the Care and Use of Laboratory Animals. The accreditation number of the ethical approval for animal experiments are SYSU‐IACUC‐2021‐001513 and SYSU‐IACUC‐2023‐001974. All Efforts were made to minimize animal suffering and to reduce the number of mice used. Some animals were excluded due to missed targets in some procedures, including the injection of viruses, the implantation of optical fiber or cannula.

### Spared Nerve Injury

The spared nerve injury (SNI) was used to establish a chronic pain and depression comorbidity model according to the previously described method.^[^ ^7a^ ^]^ Briefly, under anesthesia with isoflurane, the skin and muscle of the left thigh were incised to explore the sciatic nerve, which was composed of the common peroneal, tibial, and sural nerves. The common peroneal and tibial nerves were ligated and cut (≈2 mm sections removed), and the sural nerve was kept intact. The skin and muscle were stitched, respectively. For the sham group, three branches of sciatic nerve were exposed without injury.

### Virus and Drug Injection

The animals were fixed in a stereotactic frame (RWD Life Science Co., Shenzhen, China) with intraperitoneal injection of 1% pentobarbital sodium anesthesia. The body temperature of animals was maintained at 36 °C throughout the procedure by using a heating pad. A volume of 150 nl of virus (depending on the expression strength and viral titer) was injected by using calibrated glass microelectrodes connected to an infusion pump (KD Scientific, Holliston, MA, USA) at a slow rate of 30 nl min^−1^. The injection capillary was removed 10 min after the end of infusion to avoid virus overflow. All viruses were purchased from BrainVTA, OBiO, or Brain Case Co., Ltd. The coordinates include the following three elements: anterior–posterior (AP) from bregma, medial–lateral (ML) from the midline and dorsal–ventral (DV) from the brain surface.

For monosynaptic anterograde tracing, rAAV‐CaMKIIa‐EGFP‐WPRE‐hGH pA (AAV‐CaMKIIa‐EGFP, AAV2/9, 5.82 × 10^12^ vg ml^−1^, BrainVTA) was injected into PVP (AP: –1.25 mm; ML: +0.55 mm; DV: –2.95 mm, with a 10° angle toward the midline) or PVA (AP: –0.45 mm; ML: 0 mm; DV: –3.5 mm) of wild‐type mice to observe the location of fluorescence EGFP^+^ fiber. rAAV2/1‐hSyn‐Cre‐WPRE‐hGH pA (AAV1‐Cre, AAV2/1, 1.0 × 10^13^ vg ml^−1^, BrainVTA) was delivered into the PVP to allow the virus to spread anterogradely to the downstream VLPAG soma to express Cre (Cyclization Recombination Enzyme). Simultaneously, rAAV‐EF1a‐DIO‐hM4Di (Gi)‐mCherry‐WPRE‐hGH pA (AAV‐DIO‐hM4Di‐mCherry, AAV2/8, 5.04 × 10^12^ vg ml^−1^, BrainVTA) was injected into VLPAG (AP: –4.5 mm; ML: ±0.5 mm; DV: –2.75 mm). After 3 weeks, the mice were sacrificed under anesthesia with intraperitoneal injection of pentobarbital sodium for immunostaining with GABA antibody. Similarly, the animals, with injections into PVA with AAV1‐Cre and into NAc (AP: +1.18 mm; ML: ±1.25 mm; DV: –5 mm) with rAAV‐EF1a‐DIO‐hM3Dq (Gq)‐mCherry‐WPREs (AAV‐DIO‐hM3Dq‐mCherry, AAV2/8, 2.27 × 10^12^ vg ml^−1^, BrainVTA), were sacrificed under anesthesia with intraperitoneal injection of pentobarbital sodium for immunostaining with D1 and D2 antibodies.

For retrograde monosynaptic tracing, helper viruses that contained rAAV‐EF1a‐DIO‐H2B‐EGFP‐T2A‐TVA‐WPRE‐hGH‐pA (AAV‐DIO‐TVA‐EGFP, AAV2/9, 2.7 × 10^12^ vg ml^−1^, BrainVTA) and rAAV‐EF1a‐DIO‐oRVG‐WPRE‐hGH‐pA (AAV‐DIO‐oRVG, AAV2/9, 5.63 × 10^12^ vg ml^−1^, BrainVTA) were infused into VLPAG in GAD2‐Cre mice and NAc in D2‐Cre mice. Three weeks later, RV‐ENVA‐△G‐dsRed (2.0 × 10^8^ IFU ml^−1^, BrainVTA) was injected into the same site of VLPAG and NAc. 10 days later, the mice were sacrificed under anesthesia to observe the fluorescence dsRed^+^ signals.

For conditional induction of glutamatergic neurons apoptosis, the Cre‐dependent virus AAV‐flex‐taCasp3‐TEVp (AAV‐flex‐taCasp3, AAV2/9, 2.86 × 10^13^ vg ml^−1^, OBiO) was injected into PVP in CaMKIIa‐Cre mice. The comorbidity behaviors were performed at three weeks after injection.

The stainless‐steel guide cannulae (27G) (RWD Life Science Co., Shenzhen, China) were initially implanted 100 µm above the viral injection site into the area of interest (i.e., PVP and the PVA), as the needle (30G) for drug delivery was 100 µm longer than the cannula. Dental cement was used to secure the implant to the skull of the animal. Under the drive of a microinjection pump (KD Scientific, Holliston, MA, USA), the muscimol (MUS, 0.5 µg µl^−1^, 150 nl) (M1523, Sigma–Aldrich) or gabazine (GBZ, 0.2 µg µl^−1^, 150 nl) (SR‐95 531, Sigma–Aldrich) was separately microinjected into PVP or PVA, and kept for 2 min at the end of infusion.

### Chemogenetic Manipulation

To observe the role of PVP and PVA regions in the comorbidity, the Cre‐dependent viruses AAV‐DIO‐hM4Di/hM3Dq‐mCherry were injected into PVP or PVA of CaMKIIa‐Cre mice 21 days prior to the pre‐test. ClozapineN‐oxide (CNO, 5 mg kg^−1^) was injected intraperitoneally for 3 consecutive days for training in naïve mice. Before each test, intraperitoneal injection of CNO (5 mg kg^−1^) was administered 30 min in advance. The rAAV‐EF1a‐DIO‐mCherry‐WPRE‐hGH (AAV‐DIO‐mCherry, AAV2/9, 5.13 × 10^12^ vg ml^−1^, BrainVTA) virus was used as the control. For the circuit of PVP→VLPAG and PVA→NAc, the AAV‐DIO‐hM4Di/hM3Dq‐mCherry were injected into PVP or PVA of CaMKIIa‐Cre mice, and CNO (5 uM, 250 nl) was microinjected into the VLPAG or NAc before the behaviors test.

For the circuit of PVA^Glu^→NAc^D1^, the animals were microinjected with AAV1‐Cre into PVA and AAV‐D1‐DIO‐hM3Dq/hM4Di‐mCherry‐WPREs (AAV‐D1‐DIO‐hM3Dq‐mCherry, AAV2/9, 5.0 × 10^12^ vg ml^−1^, BrainVTA; AAV‐D1‐DIO‐hM4Di‐mCherry, AAV2/9, 5.0 × 10^12^ vg ml^−1^, BrainVTA) into NAc. CNO (5 mg kg^−1^) was injected intraperitoneally for 3 consecutive days for training in naïve mice. The behaviors tests were performed after CNO intraperitoneal treatment.

### In Vivo Optogenetic Manipulations

For optogenetics manipulation, the Cre‐dependent viruses AAV‐EF1a‐DIO‐eNpHR3.0‐mCherry‐WPRE (AAV‐DIO‐eNpHR‐mCherry, AAV2/9, 1.43 × 10^13^ vg ml^−1^, OBiO) or AAV‐EF1a‐DIO‐hChR2(H134R)‐mCherry‐WPRE (AAV‐DIO‐ChR2‐mCherry, AAV2/9, 2.18 × 10^13^ vg ml^−1^, OBiO) were injected into the PVP or PVA of CaMKIIa‐Cre mice. An optical fiber (200 mm, 0.37 NA, Inper, Hangzhou, China) was implanted on the same site. For the circuit of PVP→VLPAG and PVA→NAc, the animals were microinjected with AAV1‐Cre into PVP or PVA and AAV‐DIO‐eNpHR 3.0/ChR2‐mCherry into VLPAG or NAc, with optical fiber implanted into the VLPAG or NAc. Light stimulation patterns were delivered for 3 h per day over 3 consecutive days for training in naive mice. The comorbidity behaviors were examined with persistent photoinhibition (yellow light, 589 nm, 10 mW, constant) or photoactivation (blue light, 473 nm, 10 mW, 20 Hz, 5 ms). At the end of experiment, the location of the fibers was examined in all mice, and the data were discarded in the mice with fibers outside the target of brain regions.

### Optical‐Fiber‐Based Ca^2+^ Signal Recording—Task Stimulation Photometry

The virus of rAAV‐CaMKIIa‐GCamp6s‐WPRE (AAV‐CaMKIIa‐GCamp6s, AAV2/9, 5.33 × 10^12^ vg ml^−1^, BrainVTA) was injected into PVP or PVA in the mice implanted with optical fiber (200 µm, 0.37 NA, Inper, Hangzhou, China). Mice were habituated to the testing room for 3 days before testing. The implanted fiber was connected to the Fiber Photometry (Inper, Hangzhou, China) through a pre‐bleached optical fiber patch cord. Fluorescent indicators were excited from two excitation sources, corresponding to 470 nm (25 µW) wavelength and 410 nm (15 µW) wavelength LED light. The sampling rate was 60 (fps) and exposure time was 15 (ms). For three consecutive days, calcium signals were recorded while administering pain stimulation and aversion stimulation such as the tail suspension test (TST) and forced swimming test (FST), respectively, after a 30 min adaptation to the testing environment.

### Optical‐Fiber‐Based Ca^2+^ Signal Recording—Photostimulation Photometry

For the circuit of PVP^Glu^→VLPAG^GABA^, the rAAV‐hSyn‐DIO‐ChrimsonR‐mCherry‐WPRE‐hGH‐pA (AAV‐DIO‐ChrimsonR‐mCherry, AAV2/9, 5.71 × 10^12^ vg ml^−1^, BrainVTA) and the rAAV‐mVGAT1‐GCamp6s (AAV‐VGAT‐GCamp6s, AAV2/9, 5.52 × 10^12^ vg ml^−1^, Brain Case) were separately injected into PVP or VLPAG of CaMKIIa‐Cre mice. The optical fiber was implanted into VLPAG. Mice were habituated to the testing room for 3 days before testing. The laser transmitter (635 nm, 10 mW, 20 Hz, 5 ms) was connected to the Fiber Photometry (Inper, Hangzhou, China) through a pre‐bleached optical fiber extension cord. The implanted fiber was connected to the Fiber Photometry through a pre‐bleached optical fiber patch cord. Fluorescent indicators were excited from two excitation sources, corresponding to 470 nm (25 µW) wavelength and 410 nm (15 µW) wavelength LED light. The sampling rate was 60 (fps) and exposure time was 15 (ms). The calcium signals were obtained in the period of red‐light stimulation. For the circuit of PVA ^Glu^ → NAc ^D2^ or PVA ^Glu^ → NAc ^D1^, the virus rAAV‐CaMKIIa‐ChrimsonR‐mCherry‐WPRE‐hGH‐pA (AAV‐CaMKIIa‐ChrimsonR ‐mCherry, AAV2/9, 4.82 × 10^12^ vg ml^−1^, BrainVTA) was injected into PVA, and the virus rAAV‐DIO‐GCamp6s‐WPRE‐hGH pA (AAV‐DIO‐GCamp6s, AAV2/9, 5.14 × 10^12^ vg ml^−1^, BrainVTA) was injected into NAc of D2‐Cre or D1‐Cre mice with implantation of optical fiber.

After the calcium signals data acquisition, PloynomialFitted correction was used to reduce the photobleaching effect caused by long‐term recording. The 410 nm recorded data as a motion and bleaching control were scaled using the least‐squares regression to minimize the difference between 410 nm (calcium‐independent fluorescence) and 470 nm (calcium‐dependent fluorescence) data and subtracted from the 470 nm trajectory to generate a corrected 470 nm signal. The ΔF/F was calculated as (F‐F0)/F0, where F0 was defined as the baseline GCamp6s fluorescence signal.^[^ ^30^ ^]^ ΔF/F value was represented by heat map and average map, and the shaded area represented the standard error of average value.

### Electrophysiological Recordings—Brain Slice Preparation

Mice were deeply anesthetized with pentobarbital sodium and then intracardially perfused with ice‐cold oxygenated N‐methyl‐d‐glucamine artificial cerebrospinal fluid (NMDG ACSF) that contained the following (in mM): 93 NMDG, 20 HEPES buffer, 25 glucose, 2.5 KCl, 0.5 CaCl_2_, 1.2 NaH_2_PO_4_, 30 NaHCO_3_, 10 MgSO_4_, 5 sodium ascorbate, 3 sodium pyruvate, 2 thiourea and 3 glutathione (pH 7.3–7.4, osmolarity of 300–305 mOsmL). Coronal slices (300 µm) that included the PVP, the PVA, the NAc or the VLPAG were sectioned using a vibrating microtome (VT1200s, Leica) and were initially incubated for at least 1 h in oxygenated HEPES ACSF solution (28 °C) that contained the following (in mM): 20 HEPES, 25 glucose, 92 NaCl, 2.5 KCl, 1.2 NaH_2_PO_4_, 30 NaHCO_3_, 2 thiourea, 5 sodium ascorbate, 3 sodium pyruvate, 3 glutathione, 2 CaCl_2_ and 2 MgSO_4_ (pH 7.3–7.4, osmolarity of 300–305 mOsmL). The brain slices were then transferred to a slice chamber (Warner Instruments) for whole‐cell recording and were continuously perfused at a rate of 3 ml min^−1^ with oxygenated standard ACSF solution (32 °C) that contained the following (in mM): 3 HEPES, 10 glucose, 129 NaCl, 3 KCl, 2.4 CaCl_2_, 1.3 MgSO_4_, 1.2 KH_2_PO_4_ and 20 NaHCO_3_ (pH 7.3–7.4, osmolarity of 300–310 mOsmL). The temperature of the standard ACSF solution was maintained using an in‐line solution heater (TC‐344B, Warner Instruments).

### Whole‐Cell Patch‐Clamp Recordings

Neurons in regions of the interest were visualized using a water‐immersion objective (× 40) on an upright microscope (BX51WI, Olympus), which was equipped with interference contrast (IR/DIC) and an infrared camera connected to the video monitor. Whole‐cell patch‐clamp recordings were performed from visually identified PVP, PVA, VLPAG and NAc neurons. Patch pipettes were pulled from borosilicate glass capillaries (outer diameter of 1.5 mm, VitalSense Scientific Instruments) on a four‐stage horizontal puller (P1000, Sutter Instruments). The current‐evoked firing was recorded in current‐clamp mode using pipettes (5–7 MΩ) filled with potassium‐gluconate‐based internal resistance solution containing the following (in mM): 130 potassium gluconate, 2 MgCl_2_, 5 KCl, 0.6 EGTA, 10 HEPES, 2 Mg‐ATP and 0.3 Na‐GTP (pH 7.2, osmolality of 285–290 mOsmL). Before collecting the data, the neurons were given at least 3 min to stabilize. All recordings were performed at 28° to 30 °C. Depolarizing currents of 0 to 200 pA (500‐ms duration) were delivered in increments of 10 pA.

### CNO‐Evoked Response

To validate the hM4Di or hM3Dq function, current‐clamp recordings were conducted at a resting membrane potential from hM4Di‐mCherry or hM3Dq‐mCherry expressing PVP neurons using glass pipettes with 3‐ to 7‐megohm tip resistance when filled with an internal solution (132 mM K‐gluconate, 3 mM KCl, 10 mM Hepes, 0.5 mM EGTA, 1 mM MgCl_2_, 12 mM Na‐phosphocreatine, 4 mM Mg‐ATP, 0.5 mM Na‐GTP, and 0.2% biocytin, adjusted to pH 7.2 to 7.3 with KOH). To monitor the changes in input resistance, a −20‐pA current was injected every 20 s. After confirming the stable baseline recordings, CNO (10 µM) was bath applied for 5 min, and the membrane potential and input resistance were compared between prior to (−1 to 0 min) and following (4 to 5 min) CNO application.

### Light‐Evoked Response

Optical stimulation was performed using a laser‐through microscope positioned above the surface of the target brain region. Light power was modulated to the lowest intensity at which we could consistently evoke a postsynaptic response, up to a maximum of 10 mW. To verify the functionality of the AAV‐DIO‐ChR2‐mCherry virus, mCherry‐labeled neurons that expressed ChR2 in CaMKIIa‐Cre mice were visualized and activated with a blue laser light using 5‐Hz, 10‐Hz, and 20‐Hz stimulation protocols with a pulse width of 5 ms. In some experiments, the functional characteristics of the AAV‐DIO‐eNpHR‐mCherry virus was assessed by stimulation with a sustained yellow laser light (594 nm, 10 mW). Light‐evoked EPSCs (EPSCs) were recorded at ‐70 mV after photostimulation of ChR2‐expressing PVP^Glu^ fibers in VLPAG slices or ChR2‐expressing PVA^Glu^ fibers in NAc slices (473 nm, 5 ms). Light‐evoked IPSCs (IPSCs) were recorded at 0 mV after photostimulation of ChR2‐expressing VLPAG^GABA^ fibers in VLPAG slices or PVA^Glu^ fibers or NAc^D1^ fibers in NAc slices (473 nm, 5 ms). EPSCs or IPSCs were recorded in voltage clamp mode (holding potential, −70 mV for EPSCs and 0 mV for IPSCs) with Cs‐methanesulfonate internal solution. The latency to the evoked EPSC or IPSC was measured from the beginning of the optogenetic stimulation. Events that occurred within 10 ms of the laser stimulation were considered to be evoked responses. The evoked responses were identified on the basis of amplitude and kinetics of the response that differed from the spontaneously recorded events. APV (100 µM) were added. DNQX (20 µM) was used to confirm glutamatergic EPSCs. The GABA type A receptor antagonist BIC (10 µM) was used to confirm GABAergic IPSCs. To test whether the postsynaptic currents recorded in vLPAG or NAc neurons were elicited by direct synaptic connections, 1 µM TTX and 100 µM 4‐AP were added to ACSF. Light‐evoked currents were recorded using Igor Pro (WaveMetrics). Currents were amplified, low‐pass–filtered at 3 kHz, and digitized at 10 kHz. Only cells with series resistance <20 megaohms and input resistance > 100 megaohms were studied. Cells were excluded if input resistance changed > 15% or series resistance changed >10% over the experiment.

### Immunohistochemistry

The animals were deeply anesthetized and subjected to cardiac perfusion with 0.9% saline followed by 4% paraformaldehyde (PFA). The brains were subsequently removed and post‐fixed in 4% PFA at 4 °C overnight. After cryoprotection of the brains with 30% (w/v) sucrose, coronal or sagittal sections (20 µm) were cut on a cryostat (LEICA CM1950). For immunostaining, brain sections were washed three times in PBS and then incubated with blocking buffer (0.3% Triton X‐100, 10% donkey serum in PBS) for 1 h at room temperature. Subsequently, these slices were incubated with primary antibodies diluted with blocking solution at 4 °C overnight, followed by the corresponding fluorophore‐conjugated secondary antibodies for 2 h at room temperature. After washing with PBS, slices were cover‐slipped in mounting medium (Beyotime). Then, a Nikon (Eclipse Ni‐E, Nikon, Japan) fluorescence microscope was used to examine the sections, and a Nikon DS‐Qi2 camera was used to capture images. For primary antibodies, we used antibodies against c‐Fos (rabbit, 1:400; CST, #2250), CaMKIIa (mouse, 1:100; abcam, ab22609), GABA (rabbit, 1:800; Sigma, A2052). Alexa‐488 (mouse, rabbit, or rat, 1:200; Jackson ImmunoResearch) and Cy3 (mouse, rabbit, or rat, 1:400; Jackson ImmunoResearch) were used as the secondary antibodies.

For immunostaining with antibody Dopamine D1 Receptor (rabbit, 1:200; abcam, ab20066), Dopamine D2 Receptor (rabbit, 1:200; Sigma, AB5084P), the protocol has been improved. Briefly, the obtained coronal brain slices were washed three times in PBS and then performed antigen repair with Citrate‐EDTA Antigen Retrieval Solution (Beyotime, P0086, China) for 1 minute. After washed in PBS, brain sections were incubated with blocking buffer for 15 min at room temperature and then incubated with primary antibodies diluted with blocking solution at 4 °C overnight, and continue at room temperature for 1 h. The following steps are the same as before.

### Von Frey Test (VFT)

The mechanical withdrawal threshold was measured using the von Frey filaments. Briefly, each animal was allowed to acclimate to the testing environment for 3 consecutive days (15 mins day^−1^) before the behavior tests. Different strengths of Von‐Frey fibers were used alternately to stimulate the lateral part of the hind paw. If a withdrawal reaction did not occur, the next higher force fiber was applied. A nociceptive response was defined as a quick paw withdrawal or paw flinching following von Frey filament application. This “up and down” method defined the mechanical sensitivity that produced a 50% likelihood of withdrawal. All behavioral tests were conducted by a researcher who was blinded to the treatment conditions.

### Assessment of Depression‐Like Behaviors

All behaviors were performed as our previously described.^[^ ^7a^ ^]^ Mice was habituated to the testing room for at least 60 min on each test day. All behavioral tests were videotaped using a video tracking system. The activity behaviors were analyzed by software (Shanghai Jiliang Software Technology, Co., Ltd.). The instrument was cleaned with 75% Ethanol between the behavioral sessions.

### Open Field Test (OFT)

The mice were gently placed in the center of open‐topped white box (length, 500 mm; width, 500 mm; and height, 400 mm), which was placed in a sound‐attenuated box. The movement of mice was tracked for 6 min by using the digital video camera in a bright environment and the data were measured in the last 5 min.

### Elevated Plus Maze Test

The elevated plus maze test (EPM) apparatus, which consists of a central platform (5 × 5 cm^2^), two closed arms (35 × 5 × 10 cm^3^) and two opposing open arms (35 × 5 cm^2^), was placed 55 cm above the floor in a sound‐attenuated box. For the first time, the mice were habituated to the testing room for at least 60 min on each test day. At the start of the test, each mouse was placed in the central platform facing an open arm and was allowed to explore the maze for 6 min. The test was measured in the last 5 min.

### Sucrose Preference Test (SPT)

Mice were housed individually and habituated with two identical bottles of 1% sucrose for 2 d followed with 2 d of water. Then the experimental mice were water deprived for 24 h. Next, the mice were presented with two bottles for 2 h, one containing water and the other containing 1% sucrose, and the bottles’ positions were switched after 1 h. The consumption of each fluid was measured.

### Tail‐Suspension Test (TST)

Each animal was individually suspended ≈50 cm above the floor using adhesive tape that was placed roughly 1 cm from the tip of the tail. Each mouse was tested for 6 min. The mice's behavior was recorded with a video camera from the side, and the duration of immobility was measured in the last 5 min.

### Forced Swimming Test

Each mouse was placed in a transparent cylinder (30 cm high and 12 cm in diameter) that contained fresh water (24 ± 1 °C) up to a depth of 25 cm from the bottom. The forced swimming test (FST) was performed for 6 min by recording with a video camera and analyzed the last 5 min. The video recording was analyzed to calculate the time of immobility. Immobility was defined as the absence of all movement, except that necessary to keep the nose above water. Among all behavioral tests, the FST was generally arranged as the last one.

### Statistical Analysis

All data are expressed as the mean ± SEM and analyzed with SPSS 23.0 (SPSS, USA). The Shapiro‐Wilk test was used to check the normality of data. For data with a normal distribution, the student's *t*‐test, including two independent samples or paired samples, was used for the statistical comparisons. The data on painful behavior and electrophysiology were analyzed using two‐way repeated‐measures ANOVA. A nonparametric test (Mann‐Whitney test or Wilcoxon matched‐pairs signed rank test) was used instead when the normality test was not satisfied. The detailed statistical information in each experiment, including the sample size, measurements, comparisons, statistical methods, statistical tests, and probability values, etc., may be found in table 1 of Supporting information. Significance levels were *P* < 0.05. While no power analysis was performed, the sample size was based on the previous studies of painful behavior and pertinent neural circuit studies.

## Conflict of Interest

The authors declare no conflict of interest.

## Author Contributions

J.D., L.C.¸ C.C.L., and M.L., contributed equally to this work. W.J.X., T.X., J.F.Z. designed the experiments. J.D., L.C., C.C.L., M.L., J.Y.W., H.T.F., P.Y., X.Z.Z. performed the experiments. J.D., C.C.L., J.B.Z. acquired the data. J.D., G.Q.G., Z.K.Z., J.F.Z. analyzed the data. W.J.X., T.X. wrote the manuscript. W.J.X., J.D., L.C., F.Z., S.X.H. performed, finished the major revision.

## Supporting information

Supporting Information

## Data Availability

The data that support the findings of this study are available from the corresponding author upon reasonable request.
