# Supplementary material for: Distinct Thalamo‐Subcortical Circuits Underlie Painful Behavior and Depression‐Like Behavior Following Nerve Injury
Source: Adv Sci (Weinh). 2024 Jul 7;11(34):2401855. doi: 10.1002/advs.202401855 (PMC11425852; doi:10.1002/advs.202401855)
Supplement: Supplementary file 1 — Supporting Information [file ADVS-11-2401855-s001.docx]

**Supporting Information**

**Distinct Thalamo-Subcortical Circuits Underlie Painful Behavior and Depression-Like Behavior Following Nerve Injury**

Jie Deng^1, #^, Li Chen^1, #^, Cui-Cui Liu^2, #^, Meng Liu^3, #^, Guo-Qing Guo^4^, Jia-You Wei^1^, Jian-Bo Zhang^5^, Hai-Ting Fan^6^, Zi-Kun Zheng^7^, Pu Yan^8^, Xiang-Zhong Zhang^8^, Feng Zhou^9^, Sui-Xiang Huang^10^, Ji-Feng Zhang^4, *^, Ting Xu^1, *^, Jing-Dun Xie^11, *^, Wen-Jun Xin^1, *^

***Corresponding Authors.** E-mail: xinwj@mail.sysu.edu.cn;

**This PDF file includes:**

Figs. S1 to S11

Tables S1

**
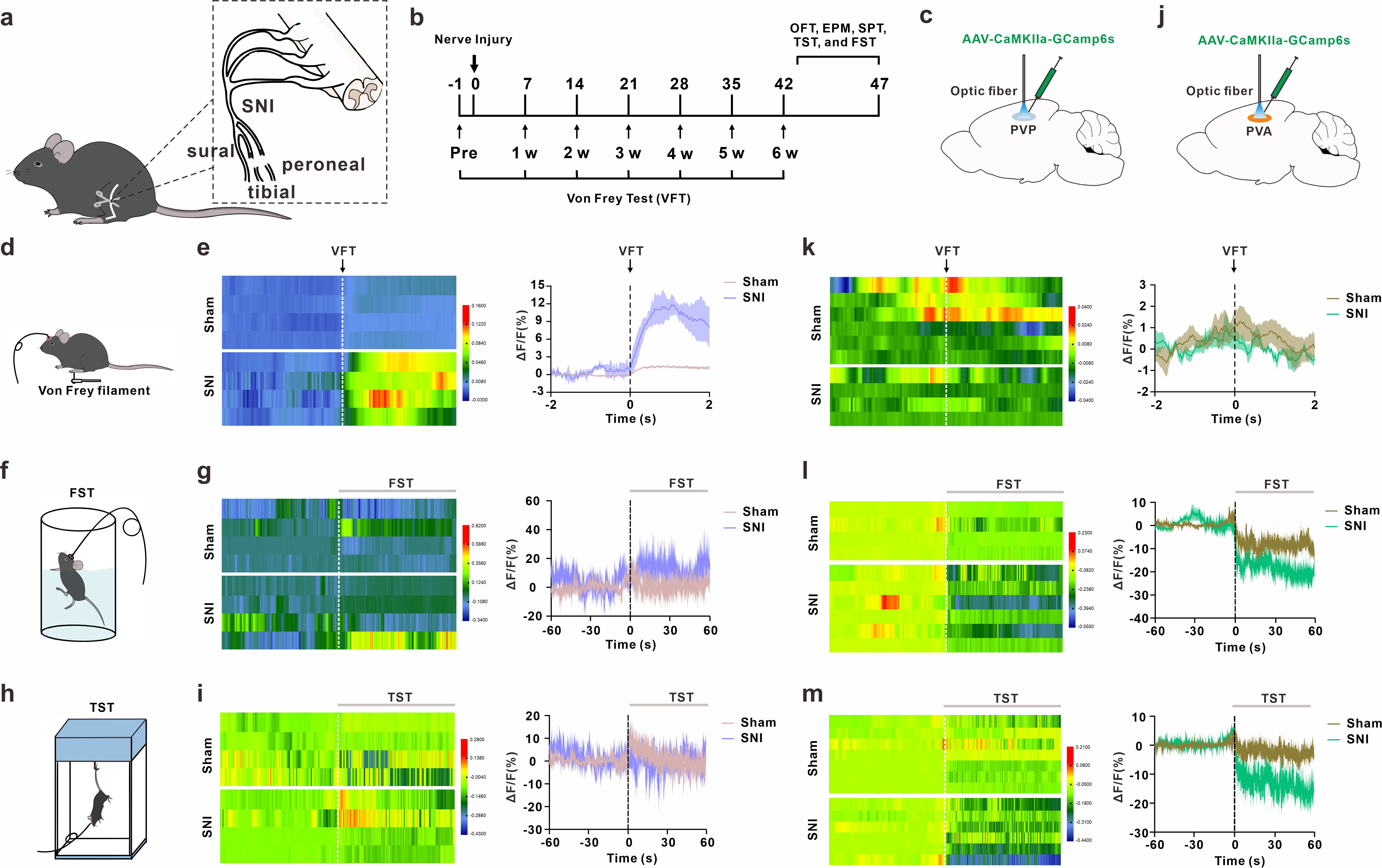
**

**Figure S1 | The activity of PVP^Glu^ or PVA^Glu^ with mechanical and aversive stimuli in naïve mice. a.** Schema of SNI surgery in mice. **b.** Schedule for experimental process. **c.** Scheme for the location of AAV-CaMKIIa-Gcamp6s injection in PVP. **d.** Schema of photometry recording with mechanical stimulation. **e.** The heatmaps (left) and the mean (right) show that the Ca^2+^ signals were significantly increased following mechanical stimuli (2g von Frey filament) in naive mice and comorbid mice (n = 4 mice for Sham group; n = 4 mice for SNI group). **f.** Schema of photometry recording with FST stimulation. **g.** The heatmaps (left) and the mean (right) showed that FST stimulation did not change the Ca^2+^ signals of PVP (n = 4 mice for the Sham group; n = 4 mice for the SNI group). **h.** Schema of photometry recording with TST stimulation. **i.** The heatmaps (left) and the mean (right) showed that TST stimulation did not change the Ca^2+^ signals of PVP (n = 4 mice for the Sham group; n = 4 mice for the SNI group). **j.** Scheme for the location of AAV-CaMKIIa-Gcamp6s injection in PVA. **k.** The heatmaps (left) and the mean (right) showed that the Ca^2+^ signals in PVA did not change following mechanical stimuli (2g von Frey filament) (n = 6 mice for the Sham group; n = 4 mice for the SNI group). **l.** The heatmaps (left) and the mean (right) show that FST stimulation decreased the Ca^2+^ signals in PVA^Glu^ in naive mice and comorbid mice (n = 4 mice for the Sham group; n = 6 mice for the SNI group). **m.** The heatmaps (left) and the mean (right) showed that TST stimulation decreased the Ca^2+^ signals in PVA^Glu^ in naive mice and comorbid mice (n = 7 mice for the Sham group; n = 6 mice for the SNI group).

*
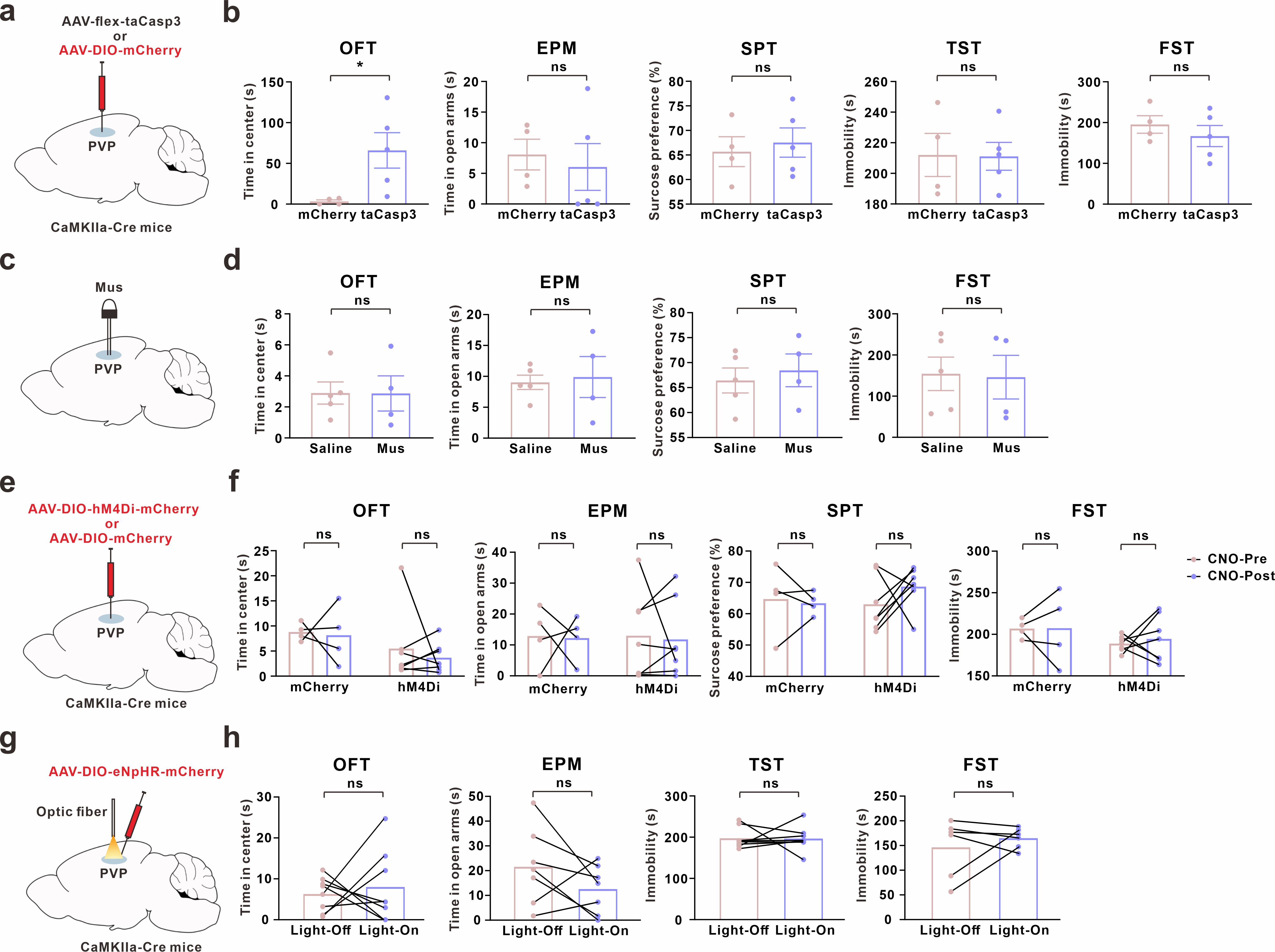
*

**Figure S2 | Inhibition activity of PVP Glu neurons did not affect depressive -like behaviors induced by SNI treatment. a.** Scheme for the injection location of AAV-flex-taCasp3 or AAV-DIO-mCherry in PVP of CaMKIIa-Cre mice. **b.** The depression-like behaviors, including EPM, SPT, TST, and FST, were not affected by the ablation of PVP neurons using AAV-flex-taCasp3 (OFT: mCherry, n=4 mice; taCasp3, n=5 mice; t(7)=2.531, p=0.0392. EPM: mCherry, n=4 mice; taCasp3, n=5 mice; t(7)=0.4175, p=0.6888. SPT: mCherry, n=4 mice; taCasp3, n=5 mice; t(7)=0.4311, p=0.6794. TST: mCherry, n=4 mice; taCasp3, n=5 mice; t(7)=0.0579, p=0.9554. FST: mCherry, n=4 mice; taCasp3, n=5 mice; t(7)=0.8153, p=0.4418).**c.** Schema for the injection location of the GABA_A_ receptor agonist muscimol (Mus). **d.** Intra-PVP injection Mus did not change the depression-like behaviors (OFT: Saline, n=5 mice; MUS, n=4 mice; t(7)=0.0203, p=0.9844. EPM: Saline, n=5 mice; MUS, n=4 mice; t(7)=0.268, p=0.7964. SPT: Saline, n=5 mice; MUS, n=4 mice; t(7)=0.5023, p=0.6309. FST: Saline, n=5 mice; MUS, n=4 mice; t(7)=0.1243, p=0.9046). **e.** Scheme for the injection location of AAV-DIO-hM4Di-mCherry or AAV-DIO-mCherry in PVP of CaMKIIa-Cre mice. **f.** Intraperitoneal injection CNO had no impact on the time spent in center of OFT, in open arms of EPM, the sucrose preference of SPT and the immobility time in FST in comorbidity mice after SNI surgery (OFT: mCherry, n=4 mice; t(3)=-0.175, p=0.872; hM4Di, n=7 mice; Z=-0.338, p=0.735. EPM: mCherry, n=4 mice; t(3)=-0.083, p=0.939; hM4Di, n=7 mice; Z=-0.507, p=0.612. SPT: mCherry, n=4 mice; t(3)=-0.295, p=0.787; hM4Di, n=7 mice; Z=-1.014, p=0.31. FST: mCherry, n=4 mice; t(3)=0.022, p=0.984; hM4Di, n=7 mice; t(6)=0.466, p=0.657.). **g.** Scheme of PVP injection with AAV-DIO-eNpHR-mCherry and implantation of optical fiber for yellow light stimulation. **h.** Photostimulation did not change the depression-like behaviors in comorbidity mice (OFT: eNpHR, n=8 mice; t(7)=0.419, p=0.687. EPM: eNpHR, n=7 mice; t(6)=-1.425, p=0.204. TST: eNpHR, n=8 mice; t(7)=-0.057, p=0.956. FST: eNpHR, n=6 mice; t(5)=0.781, p=0.47).


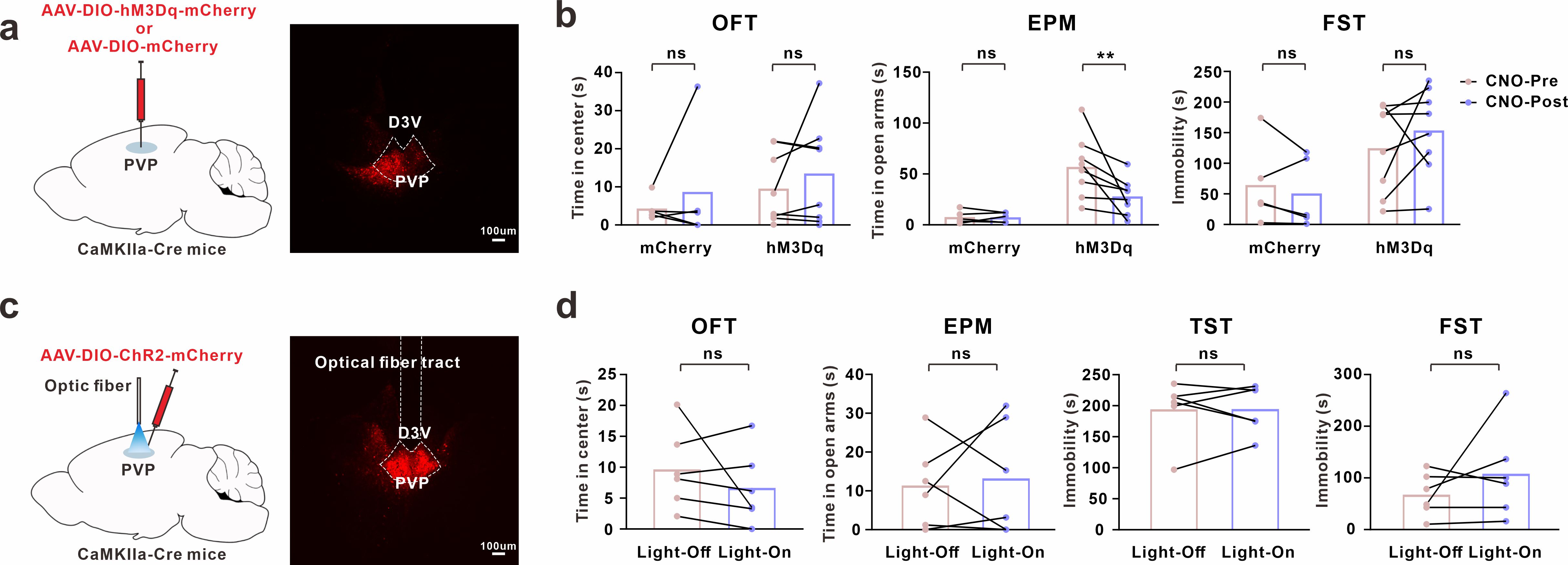


**Figure S3 | Increase of activity of PVP ^Glu^ neurons did not affect depression-like behaviors in naïve mice. a.** Scheme for the injection location of AAV-DIO-hM3Dq-mCherry or AAV-DIO-mCherry (left) and typically image of restricted fluorescence (right). Scale bar, 100 um. **b.** The depression-like behaviors, including OFT, EPM, and FST, were evaluated by intraperitoneal application CNO in both groups (OFT: mCherry, n=5 mice; Z=-0.135, p=0.893; hM3Dq, n=8 mice; Z=-0.42, p=0.674. EPM: mCherry, n=5 mice; t(4)=-0.157, p=0.883; hM3Dq, n=8 mice; Z=-2.521, p=0.012. FST: mCherry, n=5 mice; t(4)=-0.943, p=0.399; hM3Dq, n=8 mice; t(7)=1.098, p=0.309). **c.** Scheme for the injection location of AAV-DIO-ChR2-mCherry and implantation of optical fiber (left) and typical image of restricted fluorescence and optical fiber trace (right). Scale bar, 100 um. **d.** The depression-like behaviors, including OFT, EPM, TST, and FST, were evaluated before and after blue light stimulation (OFT: ChR2, n=6 mice; Z=-0.943, p=0.345. EPM: ChR2, n=6 mice; t(5)=0.315, p=0.766. TST: ChR2, n=6 mice; t(5)=0.032, p=0.976. FST: ChR2, n=6 mice; Z=-0.734, p=0.463).


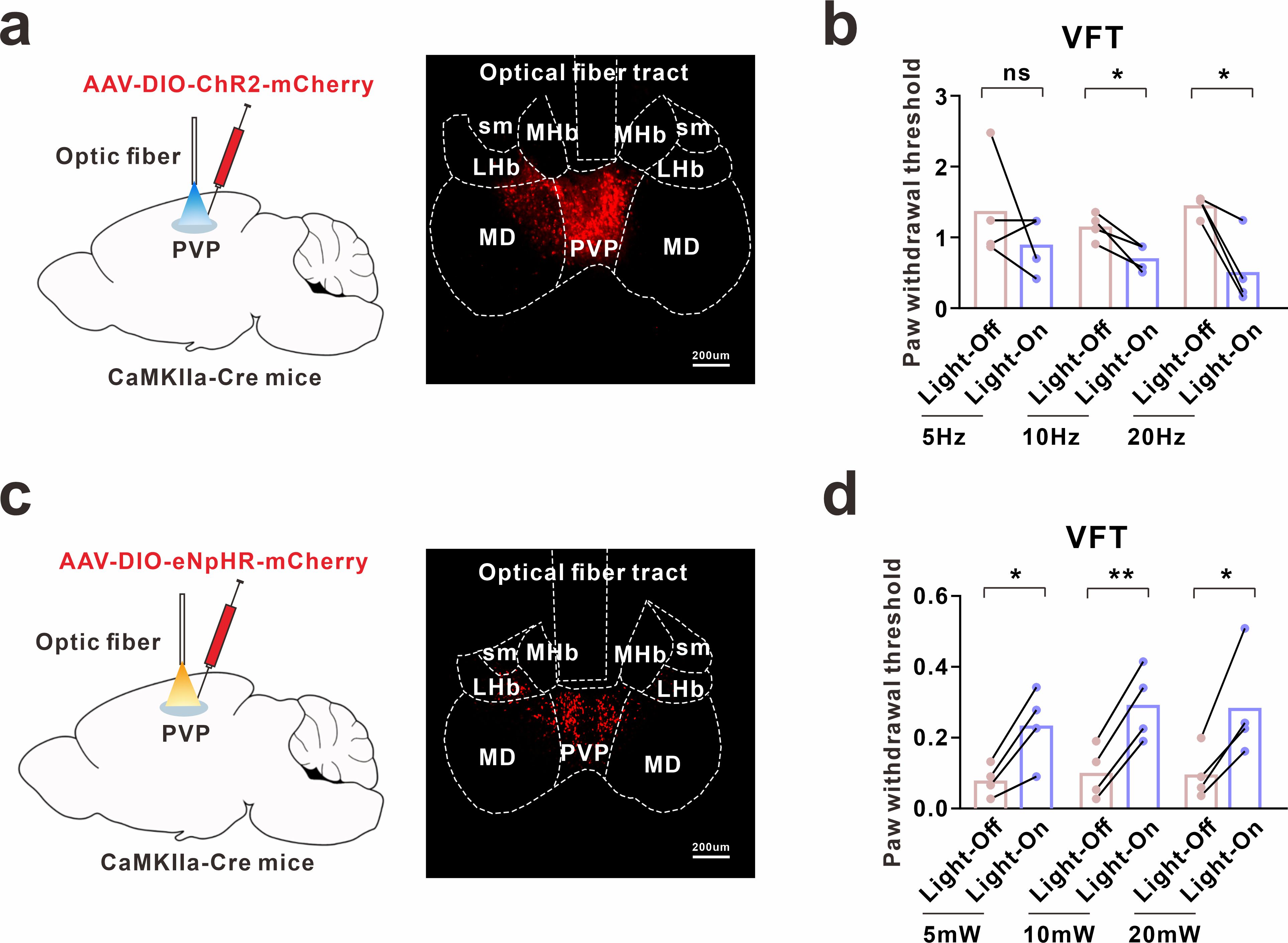


**Figure S4 | Light frequency investigation of ChR2 experiment and light power investigation of eNpHR experiment. a.** Scheme for the injection location of AAV-DIO-ChR2-mCherry and implantation of optical fiber (left) and typical image of restricted fluorescence and optical fiber trace (right). Scale bar, 200 um. **b.** The paw withdrawal threshold were performed before and after with difference frequency of blue-light stimulation (5Hz, n=4 mice; t(3)= -1.032, p=0.3779; 10Hz, n=4 mice; t(3)= -4.295, p=0.0232; 20Hz, n=4 mice; t(3)= -4.312, p=0.023). **c.** Scheme for the injection location of AAV-DIO-eNpHR-mCherry and implantation of optical fiber (left) and typical image of restricted fluorescence and optical fiber trace (right). Scale bar, 200 um. **d.** The paw withdrawal threshold were performed before and after with difference power of yellow-light stimulation (5mW, n=4 mice; t(3)= 4.773, p= 0.0175; 10mW, n=4 mice; t(3)= 13.107, p= 0.001; 20mW, n=4 mice; t(3)= 4.453, p=0.0211).


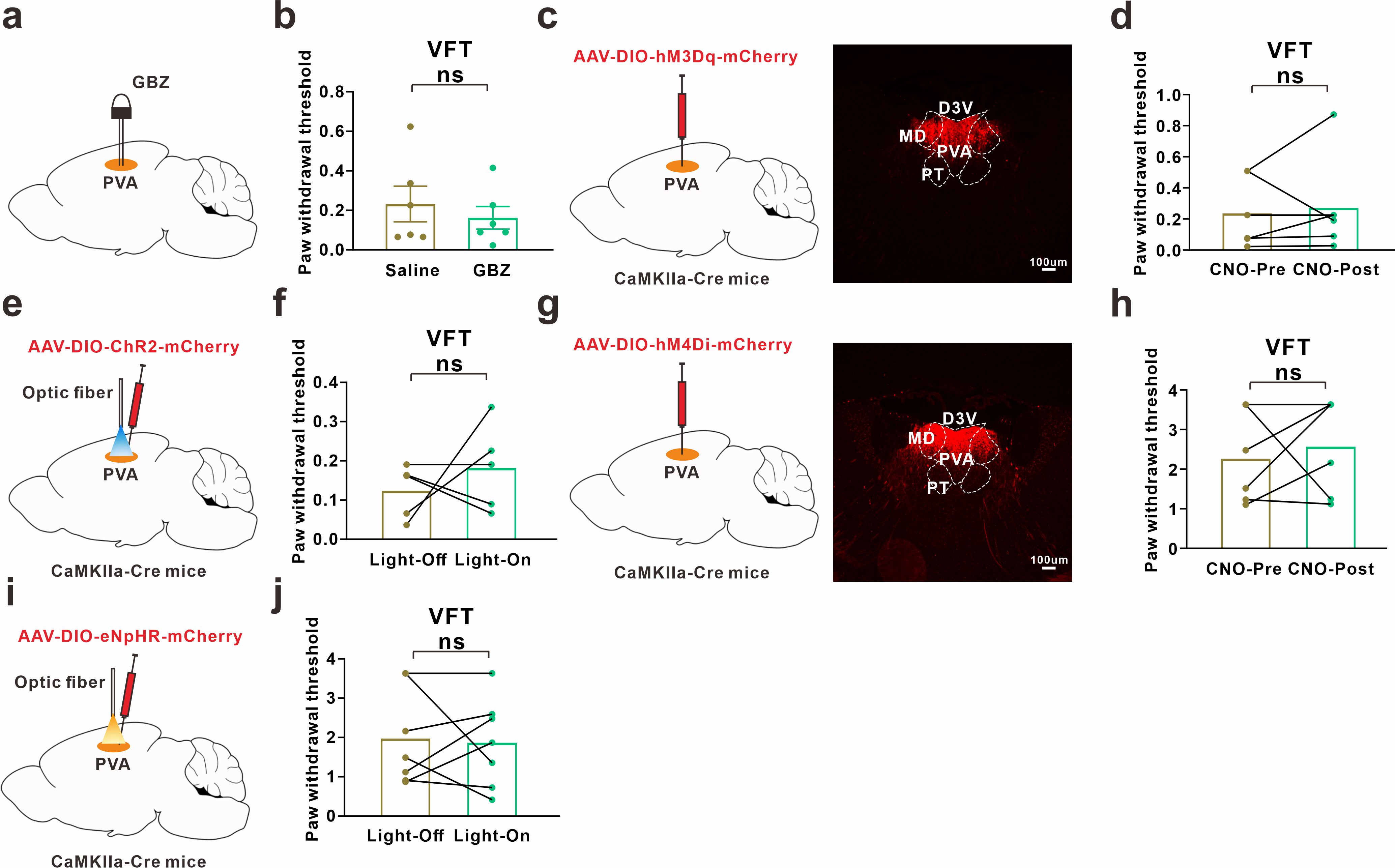


**Figure S5 | Reducing the excitability of PVA Glu neurons did not affect the paw withdrawal threshold. a.** Schema of microinjection of GBZ into PVA. **b.** Intra-PVA injection of GBZ did not affect the paw withdrawal threshold (Saline, n=6 mice; GBZ, n=6 mice; t(10)=0.6566, p=0.5263). **c.** Scheme for the injection of AAV-DIO-hM3Dq-mCherry in PVA of CaMKIIa-Cre mice (left) and typical image of restricted fluorescence (right). Scale bar, 100 um. **d.** The Von Frey test was performed before and after intraperitoneal injection of CNO (hM3Dq, n=6 mice; t(5)=0.392, p=0.712). **e.** Scheme of PVA injection with AAV-DIO-ChR2-mCherry and implantation of optical fiber for blue light stimulation. **f.** Photostimulation did not change the mechanical allodynia in comorbidity mice after SNI treatment (ChR2, n=5 mice; t(4)=0.771, p=0.484). **g.** Scheme for the injection location of AAV-DIO-hM4Di-mCherry in PVA of CaMKIIa-Cre mice (left) and typical image of restricted fluorescence and optical fiber trace (right). Scale bar, 100 um. **h.** After applying CNO, the paw withdrawal threshold remained unchanged (hM4Di, n=6 mice; t(5)=0.48, p=0.651). **i.** Scheme of PVA injection with AAV-DIO-eNpHR 3.0-mCherry and implantation of optical fiber for yellow light stimulation. **j.** Stimulation with yellow light (594 nm) did not change the paw withdrawal threshold in naïve mice (eNpHR, n=7 mice; t(6)=-0.227, p=0.828).


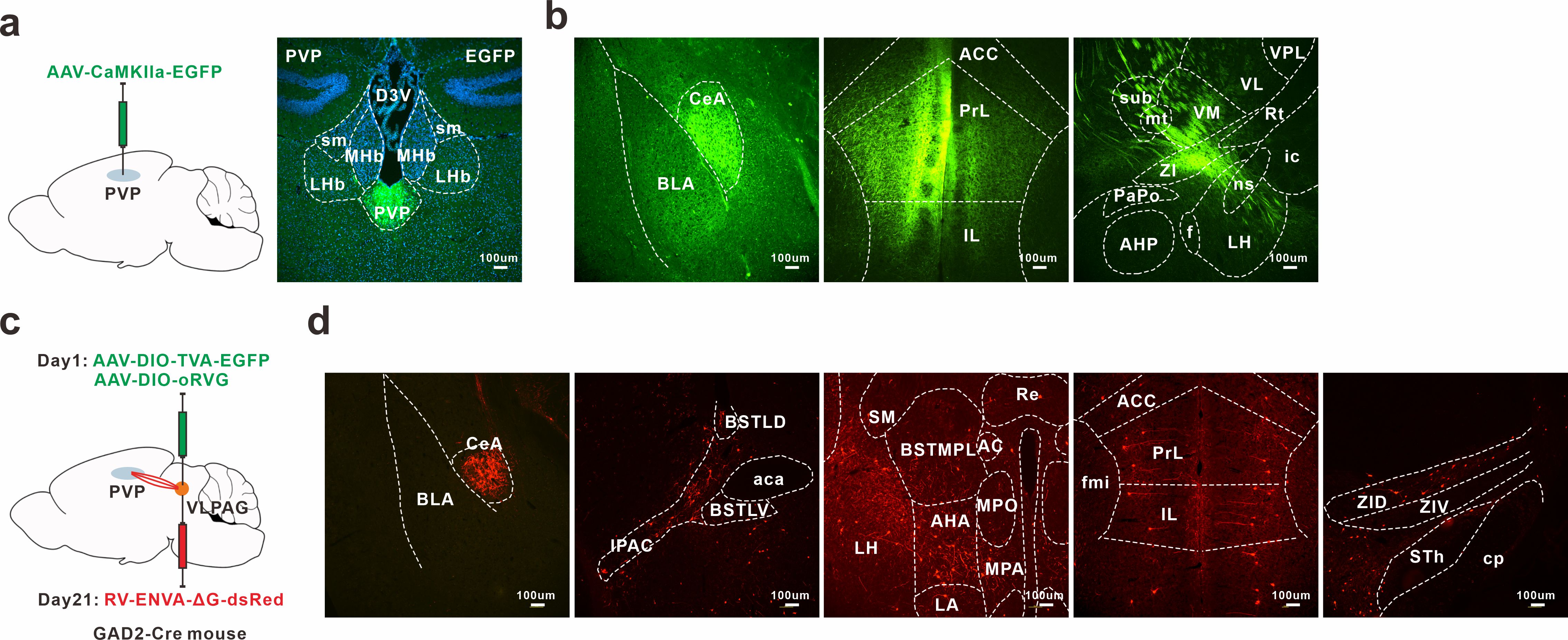


**Figure S6 | The anterograde and retrograde tracing of PVP-VLPAG circuit. a.** Schema of PVP injection of AAV-CaMKIIa-EGFP in mice (left) and representative fluorescence image of injection site in PVP (right). Scale bar, 100µm. **b.** Multiple brain regions including CeA, PrL, and ZI showed a large number of EGFP-positive fibers. Scale bar, 100µm. **c.** Schema of the Cre-dependent retrograde trans-monosynaptic RV tracing strategy in GAD2-Cre mice. **d.** Representative images showing the input from brain regions including CeA, IPAC, LH, AHA, mPFC, and ZI. Scale bar, 100 μm.


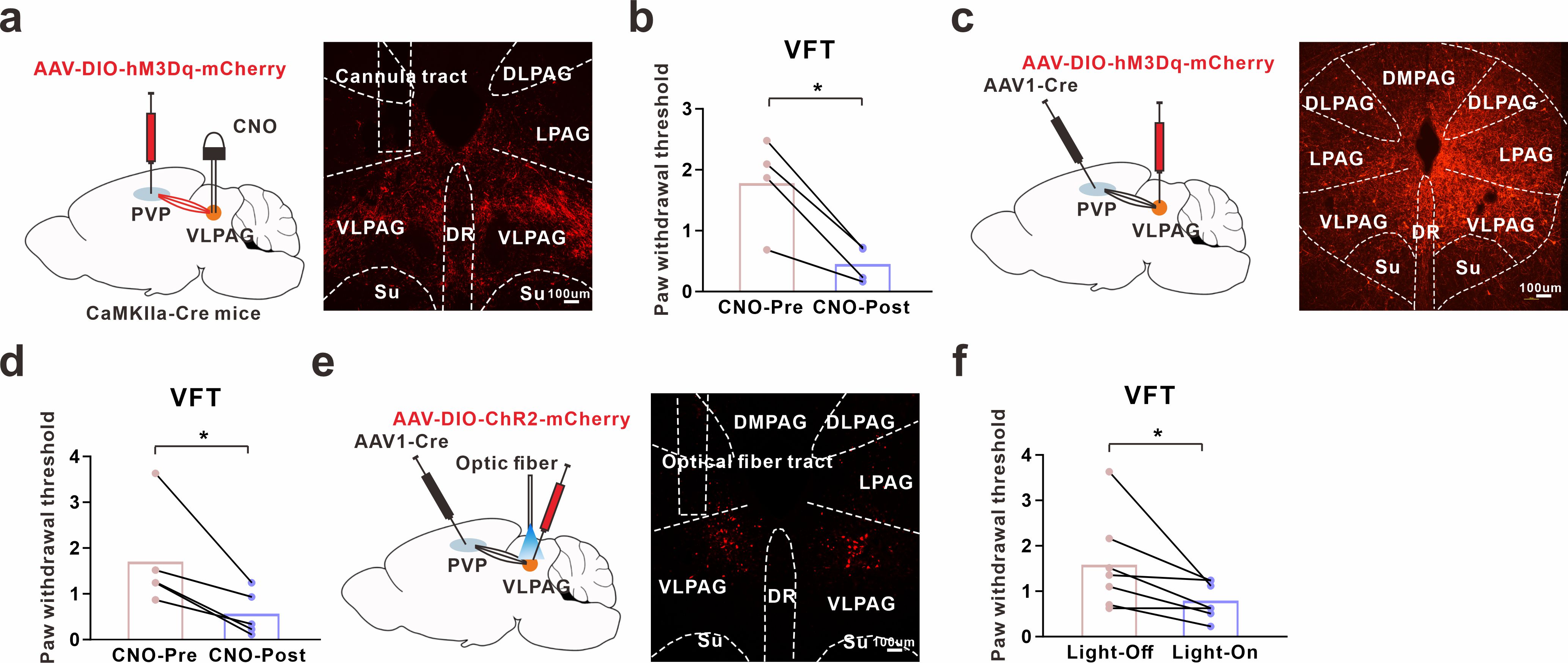


**Figure S7 | Activation of PVP Glu → VLPAG circuits induced pain conditions in naïve mice. a.** Schema of injection of AAV-DIO-hM3Dq-mCherry into PVP in CaMKIIa-Cre mice and implantation of cannula into VLPAG (left), and typical image showing the injection site in VLPAG. Scale bar, 100µm. **b.** The mechanical allodynia was induced by intraperitoneal injection of CNO in naive mice (hM3Dq, n=4 mice; t(3)=-4.725, p=0.018). **c.** Schema of injection of AAV1-hSyn-Cre into PVP and AAV-DIO-hM3Dq-mCherry into VLPAG (left), and typical image showing the injection site in VLPAG. Scale bar, 100µm. **d.** The paw withdrawal threshold was decreased by inhibition of PVP-projected VLPAG neurons with intraperitoneal application of CNO (hM3Dq, n=5 mice; t(4)=-3.39, p=0.028). **e.** Schema of injection of AAV1-hSyn-Cre into PVP and AAV-DIO-ChR2-mCherry into VLPAG and implantation of cannula into VLPAG (left), and typical image showing the injection and implantation site in VLPAG. Scale bar, 100µm. **f.** The paw withdrawal threshold was decreased by inhibition of PVP-projected VLPAG neurons with light stimulation (ChR2, n=7 mice; t(6)=-2.487, p=0.047).


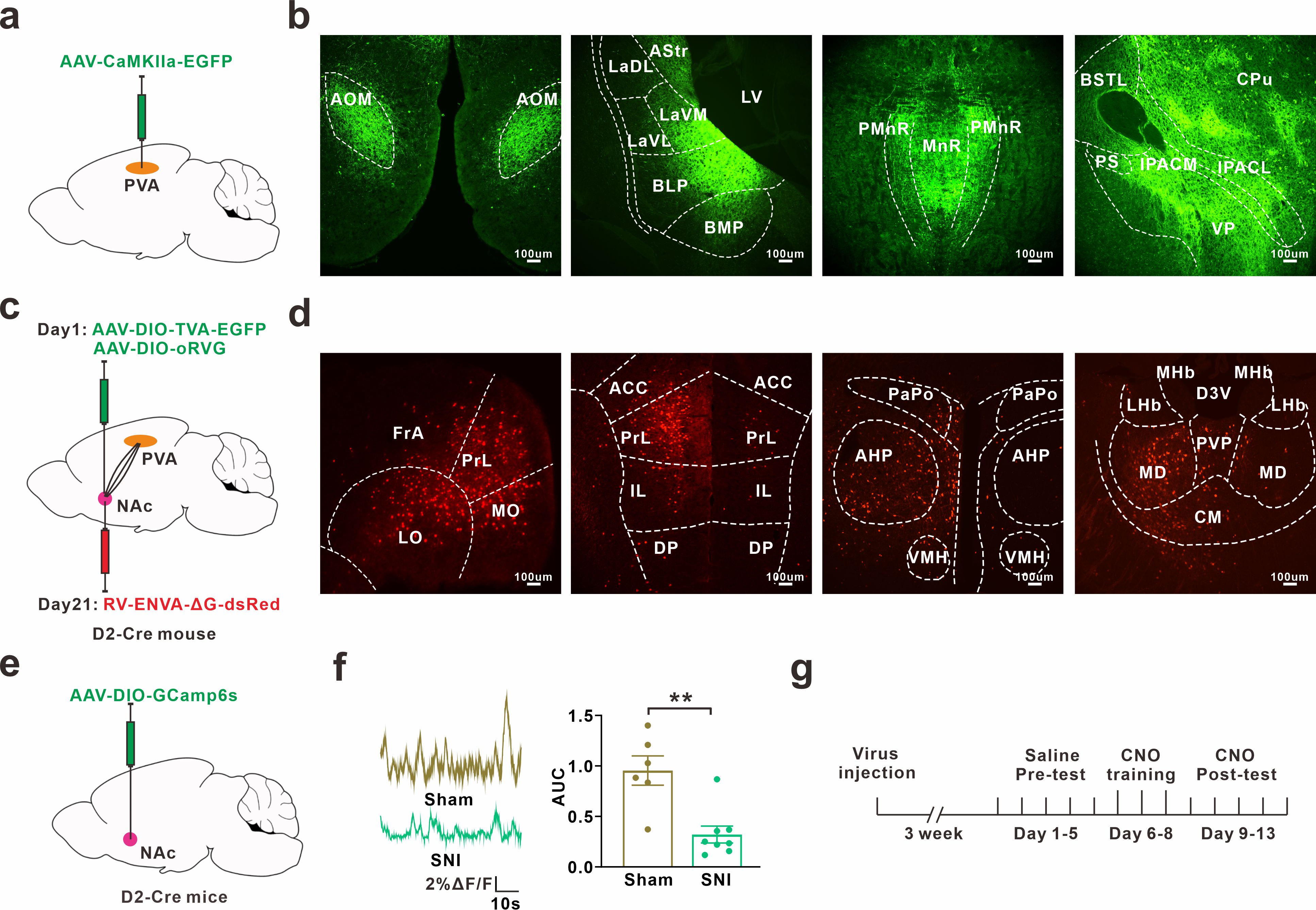


**Figure S8 | The anterograde and retrograde tracing of PVA-NAc circuit. a.** Schema of PVA injection of AAV-CaMKIIa-EGFP in mice. **b.** Representative images of EGFP+ fibers in multiple brain regions, including, AOM, LaVM, MnR and VP. Scale bar, 100 µm. **c.** Schema of the Cre-dependent retrograde trans-monosynaptic RV tracing strategy in D2-Cre mice. **d.** Representative images of dsRed-positive signals in multiple brain regions. Scale bar, 100 µm. **e.** Schema of NAc injection of AAV-DIO-GCamp6s in D2-Cre mice. **f.** Example (left) and histogram of area under the curve (AUC) (right) of fiber photometry traces from NAc^D2^ neurons in mice treated with sham or SNI (Sham, n= 6 mice; SNI, n= 8 mice; U=2, p=0.0027). **g.** Schedule of experiment in naïve mice.


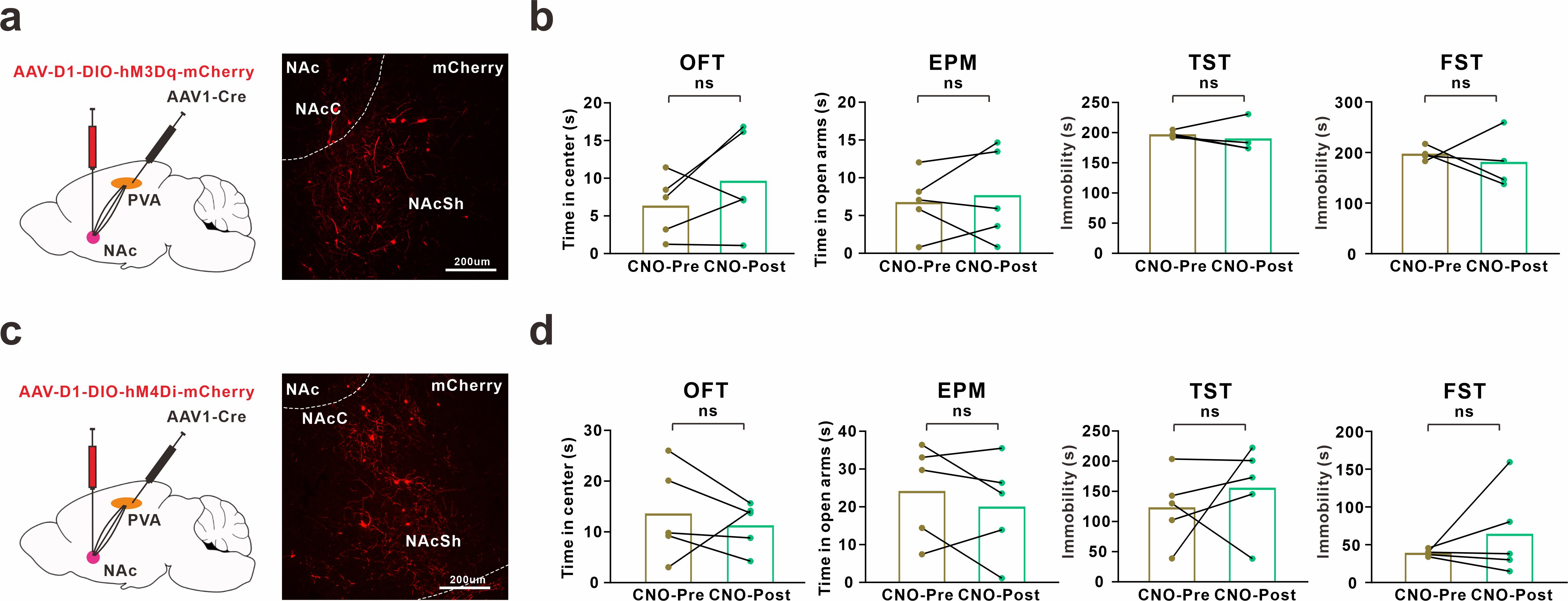


**Figure S9 | The PVA^Glu^→NAc^D1^ circuit did not have impact on depression-like behavior. a.** Schema of injection of AAV1-Cre or AAV-D1-DIO-hM3Dq-mCherry into PVP or NAc, respectively (left), and typical image of virus expression in NAc (right). Scale bar, 200*µm*. **b.** The depression-like behaviors were performed before and after CNO intraperitoneal application (OFT: hM3Dq, n=5 mice; t(4)=1.314, p=0.259. EPM: hM3Dq, n=5 mice; t(4)=0.477, p=0.659. TST: hM3Dq, n=4 mice; t(3)=-0.609, p=0.586. FST: hM3Dq, n=4 mice; t(3)=-0.479, p=0.665). **c.** Schema of injection of AAV1-Cre or AAV-D1-DIO-hM4Di-mCherry into PVP or NAc in naïve mice, respectively (left), and typical image of virus expression in NAc (right). Scale bar, 200*µm*. **d.** Continuous injection of CNO did not induce depression-like behavior in naïve mice (OFT: hM4Di, n=5 mice; t(4)=-0.633, p=0.561. EPM: hM4Di, n=5 mice; t(4)=-1.037, p=0.358. TST: hM4Di, n=5 mice; t(4)=0.731, p=0.505. FST: hM4Di, n=5 mice; t(4)=1.002, p=0.373).


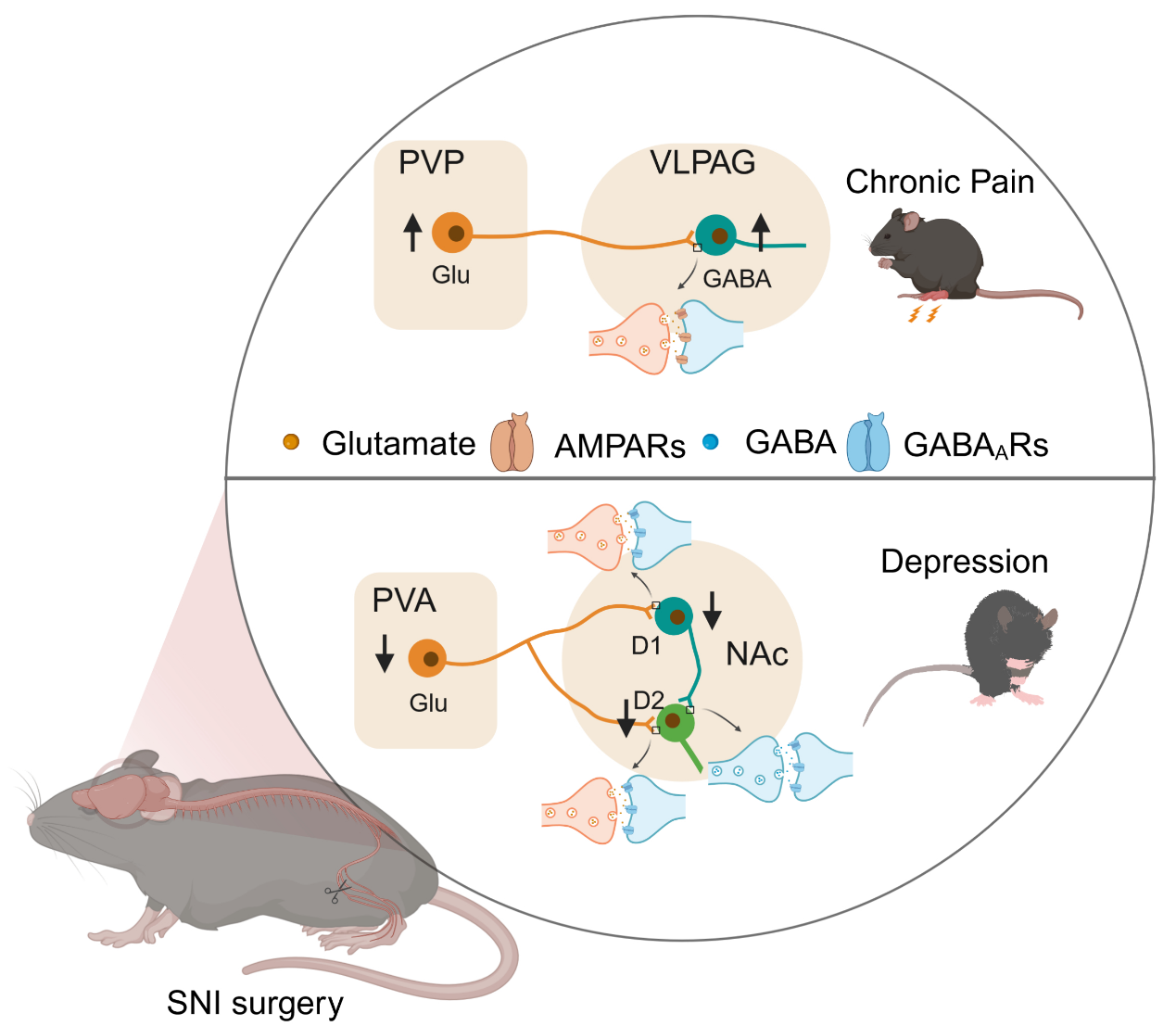


**Figure S10 | Distinct paraventricular thalamus nucleus circuits underlie the chronic pain and depression-like behavior in comorbid.** Following nerve injury, the enhanced excitatory of VLPAG^GABA^ neurons, which received the projection of PVP^Glu^, was involved in the painful behavior in comorbid. On the other hand, the circuit involving the decreased PVA^Glu^ projection to both NAc^D1^ and NAc^D2^ neurons and the net decreased activity in NAc^D2^ neurons contributed to the depression-like behavior in comorbid.


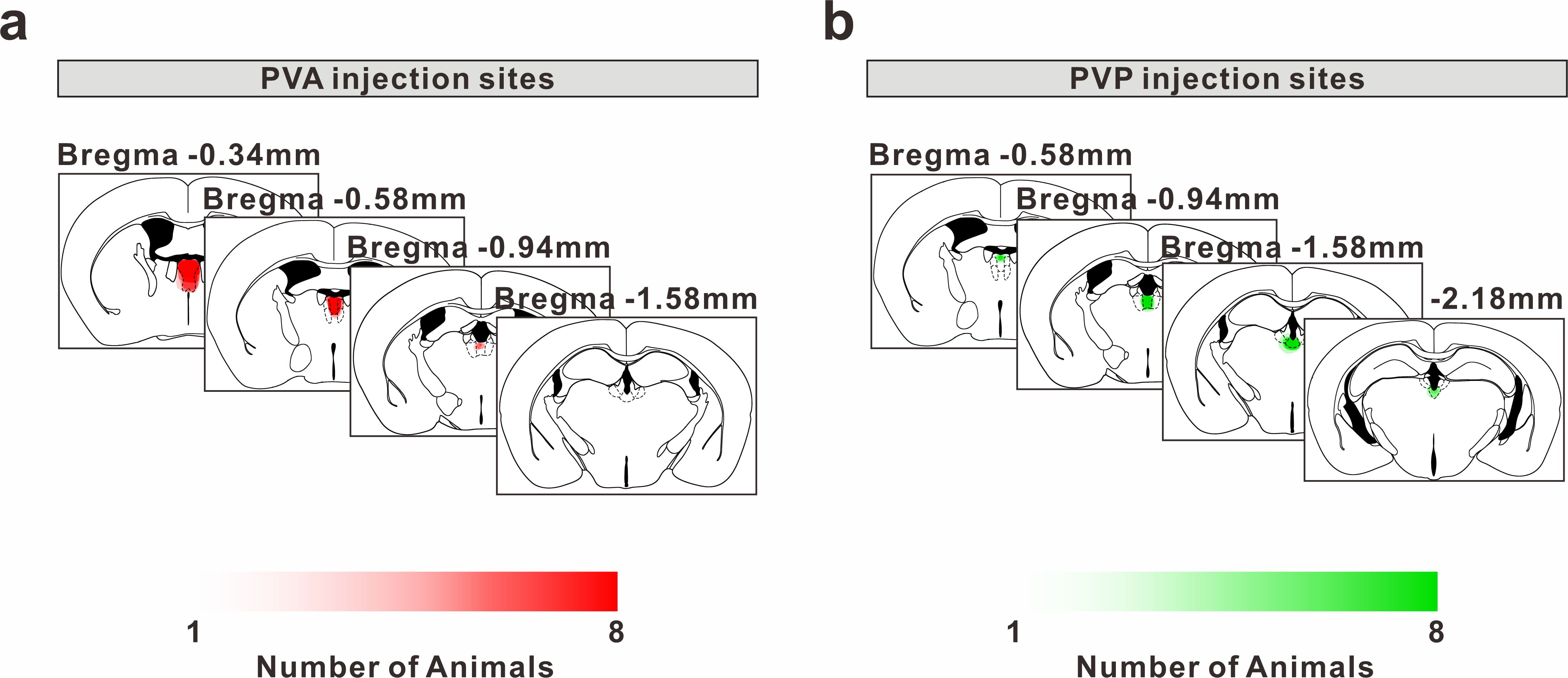


**Figure S11 | Virus expression of the experiment. a.** Schematics showing the PVA and its surrounding brain nucleus from anterior to posterior. **b.** Schematics showing the PVP and its surrounding brain nucleus from anterior to posterior.

**Table S1. Statistical information of the Figure Panel**

| **Figure** | **Amount** | **test** | **Measure** | **Comparison** | **Analysis** | **Test** | P value |
| --- | --- | --- | --- | --- | --- | --- | --- |
| 1a | Sham: 6 mice  SNI: 6 mice | Von frey test | paw withdrawal threshold | Sham vs. SNI | Two-way RM ANOVA | F(1, 10)=44.68 | p<0.0001 |
| 1b | Sham: 13 mice  SNI: 10 mice | Open field test | time spent in center | Sham vs. SNI | Mann Whitney test | U=18 | p=0.0025 |
|  | Sham: 13 mice  SNI: 13 mice | Elveated plus maze test | time spent in open arms | Sham vs. SNI | Mann Whitney test | U=3.5 | p<0.0001 |
|  | Sham: 6 mice  SNI: 9 mice | Sucrose preference test | Sucrose preference | Sham vs. SNI | Unpaired t-test (two-tailed) | t(13)=5.092 | p=0.0002 |
|  | Sham: 11 mice  SNI: 11 mice | Tail suspension test | Immobility time | Sham vs. SNI | Unpaired t-test (two-tailed) | t(20)=3.061 | p=0.0062 |
|  | Sham: 13 mice  SNI: 11 mice | Forced swimming test | Immobility time | Sham vs. SNI | Unpaired t-test (two-tailed) | t(22)=8.871 | p<0.0001 |
| 1c | Sham: 13 images  SNI: 10 images | Immunostaing | PVA cFos signals | Sham vs. SNI | Mann Whitney test | U=55 | p=0.5629 |
|  | Sham: 13 images  SNI: 10 images | Immunostaing | PVP cFos signals | Sham vs. SNI | Unpaired t-test (two-tailed) | t(21)=2.381 | p=0.0268 |
| 1g | Sham: 25 cells  SNI: 20 cells | whole-cell recording | Firing rate | Sham vs. SNI | Two-way RM ANOVA | F(1, 43)=4.968 | p=0.0311 |
| 1i | Sham: 8 mice  SNI: 8 mice | photometry | Calcium signals | Sham vs. SNI | Mann Whitney test | U=12 | p=0.0379 |
| 1k | Sham: 30 cells  SNI: 24 cells | whole-cell recording | Firing rate | Sham vs. SNI | Two-way RM ANOVA | F(1, 52)=7.361 | p=0.009 |
| 1m | Sham: 7 mice  SNI: 11 mice | photometry | Calcium signals | Sham vs. SNI | Unpaired t-test (two-tailed) | t(16)=2.833 | p=0.012 |
| 2b | SNI-mCherry: 5 mice  SNI-taCasp3: 5 mice | Von frey test | paw withdrawal threshold | mCherry vs. taCasp3 | Unpaired t-test (two-tailed) | t(8)=3.191 | p=0.0128 |
| 2d | SNI-Saline: 6 mice  SNI-Mus: 6 mice | Von frey test | paw withdrawal threshold | Saline vs. Mus | Unpaired t-test (two-tailed) | t(10)=2.321 | p=0.0427 |
| 2f | SNI-mCherry: 4 mice | Von frey test | paw withdrawal threshold | CNO-pre vs. CNO-post | Paired t-test | t(3)=0.6441 | p=0.5654 |
|  | SNI-hM4Di: 7 mice | Von frey test | paw withdrawal threshold | CNO-pre vs. CNO-post | Paired t-test | t(6)=3.383 | p=0.0148 |
| 2h | SNI-eNpHR: 8 mice | Von frey test | paw withdrawal threshold | Light-off vs. Light-on | Paired t-test | t(7)=4.373 | p=0.0033 |
| 2j | naive-mCherry: 5 mice | Von frey test | paw withdrawal threshold | CNO-pre vs. CNO-post | Paired t-test | t(4)=0.0311 | p=0.9767 |
|  | naive-hM3Dq: 8 mice | Von frey test | paw withdrawal threshold | CNO-pre vs. CNO-post | Paired t-test | t(7)=4.258 | p=0.0038 |
| 2l | naive-ChR2: 7 mice | Von frey test | paw withdrawal threshold | Light-off vs. Light-on | Paired t-test | t(6)=3.35 | p=0.0154 |
| 3b | SNI-Saline: 6 mice  SNI-GBZ: 6 mice | Open field test | time spent in center | Saline vs. GBZ | Unpaired t-test (two-tailed) | t(10)=2.498 | p=0.0316 |
|  | SNI-Saline: 6 mice  SNI-GBZ: 6 mice | Elveated plus maze test | time spent in open arms | Saline vs. GBZ | Mann Whitney test | U=3 | p=0.0152 |
|  | SNI-Saline: 5 mice  SNI-GBZ: 5 mice | Tail suspension test | Immobility time | Saline vs. GBZ | Unpaired t-test (two-tailed) | t(8)=2.932 | p=0.0189 |
|  | SNI-Saline: 6 mice  SNI-GBZ: 6 mice | Forced swimming test | Immobility time | Saline vs. GBZ | Unpaired t-test (two-tailed) | t(10)=3.935 | p=0.0028 |
| 3d | SNI-hM3Dq: 8 mice | Open field test | time spent in center | CNO-pre vs. CNO-post | Paired t-test | t(7)=3.801 | p=0.007 |
|  | SNI-hM3Dq: 8 mice | Elveated plus maze test | time spent in open arms | CNO-pre vs. CNO-post | Paired t-test | t(7)=3.343 | p=0.012 |
|  | SNI-hM3Dq: 5 mice | Sucrose preference test | Sucrose preference | CNO-pre vs. CNO-post | Paired t-test | t(4)=3.467 | p=0.026 |
|  | SNI-hM3Dq: 5 mice | Tail suspension test | Immobility time | CNO-pre vs. CNO-post | Paired t-test | t(4)=-4.117 | p=0.015 |
|  | SNI-hM3Dq: 6 mice | Forced swimming test | Immobility time | CNO-pre vs. CNO-post | Paired t-test | t(5)=-3.273 | p=0.022 |
| 3f | SNI-ChR2: 7 mice | Open field test | time spent in center | Light-off vs. Light-on | Paired t-test | t(6)=2.677 | p=0.037 |
|  | SNI-ChR2: 7 mice | Elveated plus maze test | time spent in open arms | Light-off vs. Light-on | Paired t-test | t(6)=3.387 | p=0.015 |
|  | SNI-ChR2: 8 mice | Tail suspension test | Immobility time | Light-off vs. Light-on | Paired t-test | t(7)=-4.567 | p=0.003 |
|  | SNI-ChR2: 6 mice | Forced swimming test | Immobility time | Light-off vs. Light-on | Paired t-test | t(5)=-5.746 | p=0.002 |
| 3h | naive-hM4Di: 9 mice | Open field test | time spent in center | CNO-pre vs. CNO-post | Paired t-test | t(8)=-4.361 | p=0.002 |
|  | naive-hM4Di: 9 mice | Elveated plus maze test | time spent in open arms | CNO-pre vs. CNO-post | Wilcoxon matched-pairs signed rank test | Z=-2.547 | p=0.011 |
|  | naive-hM4Di: 8 mice | Sucrose preference test | Sucrose preference | CNO-pre vs. CNO-post | Paired t-test | t(7)=-2.796 | p=0.027 |
|  | naive-hM4Di: 5 mice | Tail suspension test | Immobility time | CNO-pre vs. CNO-post | Paired t-test | t(4)=2.953 | p=0.042 |
|  | naive-hM4Di: 8 mice | Forced swimming test | Immobility time | CNO-pre vs. CNO-post | Paired t-test | t(7)=4.367 | p=0.003 |
| 3j | naive-eNpHR: 7 mice | Open field test | time spent in center | Light-off vs. Light-on | Paired t-test | t(6)=-2.957 | p=0.025 |
|  | naive-eNpHR: 7 mice | Elveated plus maze test | time spent in open arms | Light-off vs. Light-on | Paired t-test | t(6)=-2.816 | p=0.031 |
|  | naive-eNpHR: 7 mice | Tail suspension test | Immobility time | Light-off vs. Light-on | Paired t-test | t(6)=3.321 | p=0.016 |
|  | naive-eNpHR: 6 mice | Forced swimming test | Immobility time | Light-off vs. Light-on | Paired t-test | t(5)=3.336 | p=0.021 |
| 4d | Sham: 15 cells  SNI: 23 cells | whole-cell recording | Firing rate | Sham vs. SNI | Two-way RM ANOVA | F(1, 36)=5.42 | p=0.0256 |
| 4f | SNI-hM4Di: 6 mice | Von frey test | paw withdrawal threshold | CNO-pre vs. CNO-post | Paired t-test | t(5)=2.864 | p=0.035 |
| 4h | SNI-eNpHR: 6 mice | Von frey test | paw withdrawal threshold | Light-off vs. Light-on | Paired t-test | t(5)=3.375 | p=0.0198 |
| 4j | niave-hM3Dq: 5 mice | Von frey test | paw withdrawal threshold | CNO-pre vs. CNO-post | Paired t-test | t(4)=-3.276 | p=0.0306 |
| 4l | naive-ChR2: 4 mice | Von frey test | paw withdrawal threshold | Light-off vs. Light-on | Paired t-test | t(3)=-3.952 | p=0.0289 |
| 5k | SNI-mCherry: 5 mice | Von frey test | paw withdrawal threshold | CNO-pre vs. CNO-post | Wilcoxon matched-pairs signed rank test | Z=-0.365 | p=0.715 |
|  | SNI-hM4Di: 6 mice | Von frey test | paw withdrawal threshold | CNO-pre vs. CNO-post | Wilcoxon matched-pairs signed rank test | Z=-2.201 | p=0.028 |
| 5m | SNI-hM4Di: 4 mice | Von frey test | paw withdrawal threshold | CNO-pre vs. CNO-post | Paired t-test | t(3)=3.481 | p=0.04 |
| 5o | SNI-eNpHR: 6 mice | Von frey test | paw withdrawal threshold | Light-off vs. Light-on | Paired t-test | t(5)=2.802 | p=0.038 |
| 6h | Sham: 21 cells  SNI: 26 cells | whole-cell recording | Firing rate | Sham vs. SNI | Two-way RM ANOVA | F(1,45)=6.812 | p=0.0122 |
| 6j | SNI-hM3Dq: 5 mice | Open field test | time spent in center | CNO-pre vs. CNO-post | Paired t-test | t(4)=3.319 | p=0.029 |
|  | SNI-hM3Dq: 5 mice | Elveated plus maze test | time spent in open arms | CNO-pre vs. CNO-post | Paired t-test | t(4)=3.617 | p=0.022 |
|  | SNI-hM3Dq: 5 mice | Sucrose preference test | Sucrose preference | CNO-pre vs. CNO-post | Paired t-test | t(4)=3.167 | p=0.034 |
|  | SNI-hM3Dq: 5 mice | Tail suspension test | Immobility time | CNO-pre vs. CNO-post | Paired t-test | t(4)=-3.407 | p=0.027 |
|  | SNI-hM3Dq: 5 mice | Forced swimming test | Immobility time | CNO-pre vs. CNO-post | Paired t-test | t(4)=-3.169 | p=0.034 |
| 6l | SNI-ChR2: 5 mice | Open field test | time spent in center | Light-off vs. Light-on | Paired t-test | t(4)=3.913 | p=0.017 |
|  | SNI-ChR2: 5 mice | Elveated plus maze test | time spent in open arms | Light-off vs. Light-on | Paired t-test | t(4)=2.939 | p=0.042 |
|  | SNI-ChR2: 5 mice | Tail suspension test | Immobility time | Light-off vs. Light-on | Paired t-test | t(4)=-3.852 | p=0.018 |
|  | SNI-ChR2: 5 mice | Forced swimming test | Immobility time | Light-off vs. Light-on | Paired t-test | t(4)=-5.177 | p=0.007 |
| 6n | SNI-hM3Dq: 6 mice | Open field test | time spent in center | CNO-pre vs. CNO-post | Paired t-test | t(5)=-2.614 | p=0.047 |
|  | SNI-hM3Dq: 6 mice | Elveated plus maze test | time spent in open arms | CNO-pre vs. CNO-post | Paired t-test | t(5)=-2.629 | p=0.047 |
|  | SNI-hM3Dq: 6 mice | Sucrose preference test | Sucrose preference | CNO-pre vs. CNO-post | Paired t-test | t(5)=-2.971 | p=0.031 |
|  | SNI-hM3Dq: 6 mice | Tail suspension test | Immobility time | CNO-pre vs. CNO-post | Paired t-test | t(5)=4.242 | p=0.008 |
|  | SNI-hM3Dq: 6 mice | Forced swimming test | Immobility time | CNO-pre vs. CNO-post | Paired t-test | t(5)=2.849 | p=0.036 |
| 7g | SNI-AAV1-NAc-hM3Dq: 7 mice | Open field test | time spent in center | CNO-pre vs. CNO-post | Paired t-test | t(6)=4.89 | p=0.003 |
|  | SNI-AAV1-NAc-hM3Dq: 7 mice | Elveated plus maze test | time spent in open arms | CNO-pre vs. CNO-post | Paired t-test | t(6)=3.195 | p=0.019 |
|  | SNI-AAV1-NAc-hM3Dq: 7 mice | Sucrose preference test | Sucrose preference | CNO-pre vs. CNO-post | Paired t-test | t(6)=4.152 | p=0.006 |
|  | SNI-AAV1-NAc-hM3Dq: 7 mice | Tail suspension test | Immobility time | CNO-pre vs. CNO-post | Paired t-test | t(6)=-2.452 | p=0.0497 |
|  | SNI-AAV1-NAc-hM3Dq: 6 mice | Forced swimming test | Immobility time | CNO-pre vs. CNO-post | Paired t-test | t(5)=-7.865 | p=0.001 |
| 7i | SNI-AAV1-NAc-ChR2: 4 mice | Open field test | time spent in center | Light-off vs. Light-on | Paired t-test | t(3)=5.744 | p=0.0105 |
|  | SNI-AAV1-NAc-ChR2: 4 mice | Elveated plus maze test | time spent in open arms | Light-off vs. Light-on | Paired t-test | t(3)=3.419 | p=0.042 |
|  | SNI-AAV1-NAc-ChR2: 4 mice | Tail suspension test | Immobility time | Light-off vs. Light-on | Paired t-test | t(3)=-5.665 | p=0.011 |
|  | SNI-AAV1-NAc-ChR2: 4 mice | Forced swimming test | Immobility time | Light-off vs. Light-on | Paired t-test | t(3)=-10.205 | p=0.002 |
| 7k | SNI-AAV1-NAc-hM4Di: 6 mice | Open field test | time spent in center | CNO-pre vs. CNO-post | Paired t-test | t(5)=-3.512 | p=0.017 |
|  | SNI-AAV1-NAc-hM4Di: 6 mice | Elveated plus maze test | time spent in open arms | CNO-pre vs. CNO-post | Paired t-test | t(5)=-3.233 | p=0.023 |
|  | SNI-AAV1-NAc-hM4Di: 6 mice | Sucrose preference test | Sucrose preference | CNO-pre vs. CNO-post | Paired t-test | t(5)=-4.036 | p=0.01 |
|  | SNI-AAV1-NAc-hM4Di: 6 mice | Forced swimming test | Immobility time | CNO-pre vs. CNO-post | Paired t-test | t(5)=5.382 | p=0.003 |
| 8e | Sham: 18 cells  SNI: 21 cells | whole-cell recording | Firing rate | Sham vs. SNI | Two-way RM ANOVA | F(1, 37)=7.155 | p=0.0111 |
| S2b | SNI-mCherry: 4 mice  SNI-taCasp3: 5 mice | Open field test | time spent in center | mCherry vs. taCasp3 | Unpaired t-test (two-tailed) | t(7)=2.531 | p=0.0392 |
|  | SNI-mCherry: 4 mice  SNI-taCasp3: 5 mice | Elveated plus maze test | time spent in open arms | mCherry vs. taCasp3 | Unpaired t-test (two-tailed) | t(7)=0.4175 | p=0.6888 |
|  | SNI-mCherry: 4 mice  SNI-taCasp3: 5 mice | Sucrose preference test | Sucrose preference | mCherry vs. taCasp3 | Unpaired t-test (two-tailed) | t(7)=0.4311 | p=0.6794 |
|  | SNI-mCherry: 4 mice  SNI-taCasp3: 5 mice | Tail suspension test | Immobility time | mCherry vs. taCasp3 | Unpaired t-test (two-tailed) | t(7)=0.0579 | p=0.9554 |
|  | SNI-mCherry: 4 mice  SNI-taCasp3: 5 mice | Forced swimming test | Immobility time | mCherry vs. taCasp3 | Unpaired t-test (two-tailed) | t(7)=0.8153 | p=0.4418 |
| S2d | SNI-Saline: 5 mice  SNI-Mus: 4 mice | Open field test | time spent in center | Saline vs. Mus | Unpaired t-test (two-tailed) | t(7)=0.0203 | p=0.9844 |
|  | SNI-Saline: 5 mice  SNI-Mus: 4 mice | Elveated plus maze test | time spent in open arms | Saline vs. Mus | Unpaired t-test (two-tailed) | t(7)=0.268 | p=0.7964 |
|  | SNI-Saline: 5 mice  SNI-Mus: 4 mice | Sucrose preference test | Sucrose preference | Saline vs. Mus | Unpaired t-test (two-tailed) | t(7)=0.5023 | p=0.6309 |
|  | SNI-Saline: 5 mice  SNI-Mus: 4 mice | Forced swimming test | Immobility time | Saline vs. Mus | Unpaired t-test (two-tailed) | t(7)=0.1243 | p=0.9046 |
| S2f | SNI-mCherry: 4 mice | Open field test | time spent in center | CNO-pre vs. CNO-post | Paired t-test | t(3)=-0.175 | p=0.872 |
|  | SNI-hM4Di: 7 mice | Open field test | time spent in center | CNO-pre vs. CNO-post | Wilcoxon matched-pairs signed rank test | Z=-0.338 | p=0.735 |
|  | SNI-mCherry: 4 mice | Elveated plus maze test | time spent in open arms | CNO-pre vs. CNO-post | Paired t-test | t(3)=-0.083 | p=0.939 |
|  | SNI-hM4Di: 7 mice | Elveated plus maze test | time spent in open arms | CNO-pre vs. CNO-post | Wilcoxon matched-pairs signed rank test | Z=-0.507 | p=0.612 |
|  | SNI-mCherry: 4 mice | Sucrose preference test | Sucrose preference | CNO-pre vs. CNO-post | Paired t-test | t(3)=-0.295 | p=0.787 |
|  | SNI-hM4Di: 7 mice | Sucrose preference test | Sucrose preference | CNO-pre vs. CNO-post | Wilcoxon matched-pairs signed rank test | Z=-1.014 | p=0.31 |
|  | SNI-mCherry: 4 mice | Forced swimming test | Immobility time | CNO-pre vs. CNO-post | Paired t-test | t(3)=0.022 | p=0.984 |
|  | SNI-hM4Di: 7 mice | Forced swimming test | Immobility time | CNO-pre vs. CNO-post | Paired t-test | t(6)=0.466 | p=0.657 |
| S2h | SNI-eNpHR: 8 mice | Open field test | time spent in center | Light-off vs. Light-on | Paired t-test | t(7)=0.419 | p=0.687 |
|  | SNI-eNpHR: 7 mice | Elveated plus maze test | time spent in open arms | Light-off vs. Light-on | Paired t-test | t(6)=-1.425 | p=0.204 |
|  | SNI-eNpHR: 8 mice | Tail suspension test | Immobility time | Light-off vs. Light-on | Paired t-test | t(7)=-0.057 | p=0.956 |
|  | SNI-eNpHR: 6 mice | Forced swimming test | Immobility time | Light-off vs. Light-on | Paired t-test | t(5)=0.781 | p=0.47 |
| S3b | naive-mCherry: 5 mice | Open field test | time spent in center | CNO-pre vs. CNO-post | Wilcoxon matched-pairs signed rank test | Z=-0.135 | p=0.893 |
|  | naive-hM3Dq: 8 mice | Open field test | time spent in center | CNO-pre vs. CNO-post | Wilcoxon matched-pairs signed rank test | Z=-0.42 | p=0.674 |
|  | naive-mCherry: 5 mice | Elveated plus maze test | time spent in open arms | CNO-pre vs. CNO-post | Paired t-test | t(4)=-0.157 | p=0.883 |
|  | naive-hM3Dq: 8 mice | Elveated plus maze test | time spent in open arms | CNO-pre vs. CNO-post | Wilcoxon matched-pairs signed rank test | Z=-2.521 | p=0.012 |
|  | naive-mCherry: 5 mice | Forced swimming test | Immobility time | CNO-pre vs. CNO-post | Paired t-test | t(4)=-0.943 | p=0.399 |
|  | naive-hM3Dq: 8 mice | Forced swimming test | Immobility time | CNO-pre vs. CNO-post | Paired t-test | t(7)=1.098 | p=0.309 |
| S3d | naive-ChR2: 6 mice | Open field test | time spent in center | Light-off vs. Light-on | Wilcoxon matched-pairs signed rank test | Z=-0.943 | p=0.345 |
|  | naive-ChR2: 6 mice | Elveated plus maze test | time spent in open arms | Light-off vs. Light-on | Paired t-test | t(5)=0.315 | p=0.766 |
|  | naive-ChR2: 6 mice | Tail suspension test | Immobility time | Light-off vs. Light-on | Paired t-test | t(5)=0.032 | p=0.976 |
|  | naive-ChR2: 6 mice | Forced swimming test | Immobility time | Light-off vs. Light-on | Wilcoxon matched-pairs signed rank test | Z=-0.734 | p=0.463 |
| S4b | 5 Hz: 4 mice | Von frey test | paw withdrawal threshold | Light-off vs. Light-on | Paired t-test | t(3)=-1.032 | p=0.3779 |
|  | 10 Hz: 4 mice | Von frey test | paw withdrawal threshold | Light-off vs. Light-on | Paired t-test | t(3)=-4.295 | p=0.0232 |
|  | 20 Hz: 4 mice | Von frey test | paw withdrawal threshold | Light-off vs. Light-on | Paired t-test | t(3)=-4.312 | p=0.023 |
| S4d | 5 mW: 4 mice | Von frey test | paw withdrawal threshold | Light-off vs. Light-on | Paired t-test | t(3)=4.773 | p=0.0175 |
|  | 10 mW: 4 mice | Von frey test | paw withdrawal threshold | Light-off vs. Light-on | Paired t-test | t(3)=13.107 | p=0.001 |
|  | 20 mW: 4 mice | Von frey test | paw withdrawal threshold | Light-off vs. Light-on | Paired t-test | t(3)=4.453 | p=0.0211 |
| S5b | SNI-Saline: 6 mice  SNI-GBZ: 6 mice | Von frey test | paw withdrawal threshold | Saline vs. GBZ | Unpaired t-test (two-tailed) | t(10)=0.6566 | p=0.5263 |
| S5d | SNI-hM3Dq: 6 mice | Von frey test | paw withdrawal threshold | CNO-pre vs. CNO-post | Paired t-test | t(5)=0.392 | p=0.712 |
| S5f | SNI-ChR2: 5 mice | Von frey test | paw withdrawal threshold | Light-off vs. Light-on | Paired t-test | t(4)=0.771 | p=0.484 |
| S5h | naive-hM4Di: 6 mice | Von frey test | paw withdrawal threshold | CNO-pre vs. CNO-post | Paired t-test | t(5)=0.48 | p=0.651 |
| S5j | naive-eNpHR: 7 mice | Von frey test | paw withdrawal threshold | Light-off vs. Light-on | Paired t-test | t(6)=-0.227 | p=0.828 |
| S7b | naive-hM3Dq: 4 mice | Von frey test | paw withdrawal threshold | CNO-pre vs. CNO-post | Paired t-test | t(3)=-4.725 | p=0.018 |
| S7d | naive-hM3Dq: 5 mice | Von frey test | paw withdrawal threshold | CNO-pre vs. CNO-post | Paired t-test | t(4)=-3.39 | p=0.028 |
| S7f | naive-ChR2: 7 mice | Von frey test | paw withdrawal threshold | Light-off vs. Light-on | Paired t-test | t(6)=-2.487 | p=0.047 |
| S8f | Sham: 6 mice  SNI: 8 mice | photometry | Calcium signals | Sham vs. SNI | Mann Whitney test | U=2 | p=0.0027 |
| S9b | SNI-hM3Dq: 5 mice | Open field test | time spent in center | CNO-pre vs. CNO-post | Paired t-test | t(4)=1.314 | p=0.259 |
|  | SNI-hM3Dq: 5 mice | Elveated plus maze test | time spent in open arms | CNO-pre vs. CNO-post | Paired t-test | t(4)=0.477 | p=0.659 |
|  | SNI-hM3Dq: 4 mice | Tail suspension test | Immobility time | CNO-pre vs. CNO-post | Paired t-test | t(3)=-0.609 | p=0.586 |
|  | SNI-hM3Dq: 4 mice | Forced swimming test | Immobility time | CNO-pre vs. CNO-post | Paired t-test | t(3)=-0.479 | p=0.665 |
| S9d | naive-hM4Di: 5 mice | Open field test | time spent in center | CNO-pre vs. CNO-post | Paired t-test | t(4)=-0.633 | p=0.561 |
|  | naive-hM4Di: 5 mice | Elveated plus maze test | time spent in open arms | CNO-pre vs. CNO-post | Paired t-test | t(4)=-1.037 | p=0.358 |
|  | naive-hM4Di: 5 mice | Tail suspension test | Immobility time | CNO-pre vs. CNO-post | Paired t-test | t(4)=0.731 | p=0.505 |
|  | naive-hM4Di: 5 mice | Forced swimming test | Immobility time | CNO-pre vs. CNO-post | Paired t-test | t(4)=1.002 | p=0.373 |
